# AI/ML advances in non-small cell lung cancer biomarker discovery

**DOI:** 10.3389/fonc.2023.1260374

**Published:** 2023-12-11

**Authors:** Minal Çalışkan, Koichi Tazaki

**Affiliations:** ^1^Translational Science Department, Precision Medicine Function, Daiichi Sankyo, Inc., Basking Ridge, NJ, United States; ^2^Translational Science Department I, Precision Medicine Function, Daiichi Sankyo, Tokyo, Japan

**Keywords:** NSCLC, lung adenocarcacinoma, machine learning, biomarkers, non-small cell lung cancer

## Abstract

Lung cancer is the leading cause of cancer deaths among both men and women, representing approximately 25% of cancer fatalities each year. The treatment landscape for non-small cell lung cancer (NSCLC) is rapidly evolving due to the progress made in biomarker-driven targeted therapies. While advancements in targeted treatments have improved survival rates for NSCLC patients with actionable biomarkers, long-term survival remains low, with an overall 5-year relative survival rate below 20%. Artificial intelligence/machine learning (AI/ML) algorithms have shown promise in biomarker discovery, yet NSCLC-specific studies capturing the clinical challenges targeted and emerging patterns identified using AI/ML approaches are lacking. Here, we employed a text-mining approach and identified 215 studies that reported potential biomarkers of NSCLC using AI/ML algorithms. We catalogued these studies with respect to BEST (Biomarkers, EndpointS, and other Tools) biomarker sub-types and summarized emerging patterns and trends in AI/ML-driven NSCLC biomarker discovery. We anticipate that our comprehensive review will contribute to the current understanding of AI/ML advances in NSCLC biomarker research and provide an important catalogue that may facilitate clinical adoption of AI/ML-derived biomarkers.

## Introduction

Lung cancer is the leading cause of cancer deaths among both men and women (1), representing approximately 25% of cancer deaths each year (2). Lung cancer is divided into two main histological subtypes: small-cell lung cancer (SCLC) and non-small cell lung cancer (NSCLC). NSCLC constitutes approximately 85% of all lung cancer cases and is the focus of our study. The treatment landscape of NSCLC is rapidly evolving due to progress in biomarker-driven targeted therapies. Mutations in 11 genes (*EGFR*, *KRAS*, *ALK*, *ROS1*, *BRAF*, *NTRK1*, *NTRK2*, *NTRK3*, *MET*, *RET, ERBB2*) have been reported as FDA-recognized biomarkers predicting patients’ response to targeted therapies. Similarly, IHC (Immunohistochemistry) quantified PD-L1 (*CD274*) expression, microsatellite instability, and Tumor Mutation Burden (TMB) have been used in clinical settings to assess whether NSCLC patients could benefit from Immune Checkpoint Inhibitor (ICIs) (**Table S1A**).

Biomarkers are being used at an ever-increasing rate to predict disease risk, prognosis, and treatment response. Several national and international efforts have been established to standardize and catalogue disease biomarkers. BEST (Biomarkers, EndpointS, and other Tools), a joint task force between the FDA and NIH, was formed to standardize biomarker definitions in different contexts of clinical use (3). The EDRN (Early Detection Research Network) catalogues biomarkers that may improve detection of early-stage cancers (4). The Pharmacogenomics Knowledgebase (PharmGKB) curates the impact of genetic variation on drug response and catalogues pharmacogenetic biomarkers (5). The FDA regulates and catalogues pharmacogenomic biomarkers in drug labeling (6). Resources such as OncoKB, COSMIC, ClinVar, and ICGC (incorporating TCGA and Cancer Genome Project data) provide prevalence information and clinical significance assertions for genetic biomarkers in cancer (7–10). The My Cancer Genome from Vanderbilt University (11) offers an integrative database summarizing the potential clinical impact of genetic as well as protein expression and genomic instability biomarkers. Similarly, TCIA (The Cancer Imaging Archive) and IBSI (Imaging Biomarker Standardization Initiative) were formed to curate and standardize image biomarkers (12, 13). Professional societies such as the NCCN (National Comprehensive Cancer Network), ESMO (European Society for Medical Oncology), ASCO (American Society of Clinical Oncology), CAP (College of American Pathologists), IASLC (International Association for the Study of Lung Cancer), and AMP (Association for Molecular Pathology) provide clinical guideline recommendations for disease biomarker testing to help improve diagnosis and selection of targeted therapies.

These important efforts contribute to improving the delivery of personalized treatment decisions. Advancements in targeted treatments in the last 20 years have improved survival of NSCLC patients with actionable biomarkers (14). However, the long-term survival rate of NSCLC is still poor with an overall relative 5-year survival rate of less than 20% (15). Clinically utilized biomarkers for NSCLC were identified using traditional statistical approaches and are currently assumed to be mutually exclusive in therapeutic decision-making. However, there is growing evidence showing that actionable biomarkers of NSCLC can co-occur within the same patient’s tumor (16–18) and it is crucial to evaluate both linear and non-linear effects of the disease biomarkers. In this regard, machine learning algorithms promise more flexible model building and the ability to recognize non-linear, complex patterns in high dimensional datasets. Notably, several AI/ML-enabled medical devices have been FDA-approved and are being used in clinical settings for automated tissue segmentation (i.e., the use of computer algorithms to identify and distinguish different structures within medical images) and feature extraction (i.e., identification of specific patterns from the medical images to aid in diagnosis) from lung CT (Computed Tomography) images (19). Similarly, several deep learning approaches have been developed to aid whole slide image analysis (20, 21) and promise to enable enhanced performance in digital pathology workflows.

Overall, machine learning algorithms have made noteworthy contributions to NSCLC diagnostic workflows and promise growing applications in biomarker discovery. In this study, we sought to evaluate trends in AI/ML applications in NSCLC biomarker research. Using a text-mining approach, we identified 215 studies that reported potential biomarkers of NSCLC using AI/ML algorithms. We catalogued these studies with respect to BEST (3) biomarker sub-types and summarized emerging patterns and trends in AI/ML-driven NSCLC biomarker discovery We emphasize that our focus in this study was to compile potential use-cases for AI/ML in NSCLC biomarker research. Therefore, we did not capture the model performance metrics of the studies we reviewed, nor did we appraise the validity of the prediction models. For quantitative sources on AI/ML model appraisal, we recommend the readers to refer to guidelines such as CHARMS (22), MLP-BIOM (23), TRIPOD (24), and PROBAST (25).

## Methods

### NSCLC terms

**Figure 1:**
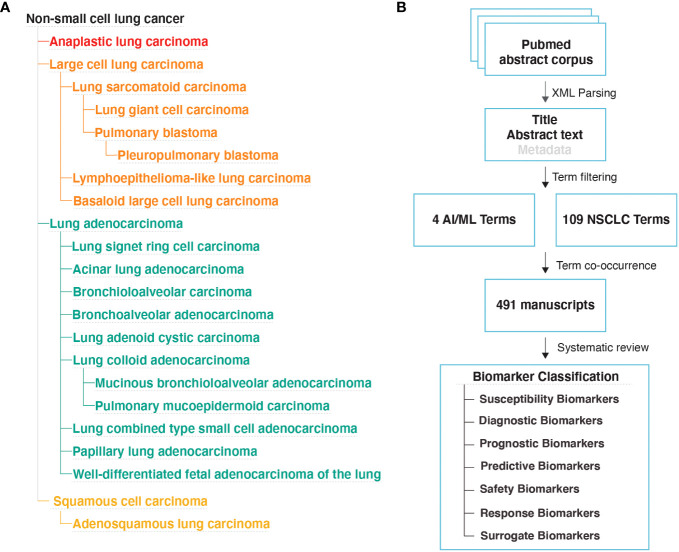
Literature Review Methodology **(A)** NSCLC disease subtypes based on the EMBL-EBI Experimental Factor Ontology (EFO) database. **(B)** Study design used to identify manuscripts that implemented AI/ML algorithms to discover new biomarkers for NSCLC.

We downloaded the EMBL-EBI Experimental Factor Ontology (EFO) (26) obo file on April 28th, 2022. We extracted all disease IDs under the EFO:0003060; non-small cell lung cancer disease category. A total of 22 NSCLC sub-types (**Figure 1A**) and 85 additional synonymous disease IDs were present in the EFO dataset, which collectively formed the NSCLC terms category (**Table S1B**).

### AI/ML terms

We used the following four terms to represent the AI/ML terms category: machine learning, artificial intelligence, deep learning, neural network.

### Text mining strategy

We downloaded MEDLINE/PubMed abstracts in xml format from the National Library of Medicine on May 4th, 2022. We used custom Python scripts to extract PubMed IDs from abstracts that include at least one of the NSCLC and at least one of the AI/ML terms. This approach (**Figure 1B**) identified a total of 491 articles that likely contain the findings of AI/ML studies on NSCLC.

### Literature review approach

We reviewed each of the 491 manuscripts and excluded 31 manuscripts that were not classified as a research article or were not written in English. We evaluated the remaining 460 manuscripts and identified 215 that reported AI/ML models that were developed to identify NSCLC biomarkers.

Specifically, we required that the models incorporated at least one data type aligning with the BEST Glossary (3) biomarker definition (i.e., a molecular, histologic, radiographic, or physiologic characteristic that is measured as an indicator of normal biological processes, pathogenic processes, or biological responses to an exposure or intervention). Across the 215 studies, we were able to categorize biomarker data types into four broad groups: (i) Molecular Biomarkers (e.g., gene expression, genotype, DNA methylation), (ii) Histologic Biomarkers (e.g., Whole Slide Image, Cytology microphotographs), (iii) Radiologic Biomarkers (e.g., Computed Tomography (CT), Magnetic Resonance Imaging (MRI), PET/CT (Positron Emission Tomography/Computed Tomography)), and (iv) Multimodal Biomarkers (i.e., a combination of different modes or types of data).

We also required that the outcome of the AI/ML models can be categorized under one of the following seven biomarker categories: (i) Susceptibility/Risk, (ii) Diagnostic, (iii) Prognostic, (iv) Predictive, (v) Response, (vi) Safety, and (vii) Surrogate. The first six biomarker categories were defined based on the BEST Glossary (3) definitions. The last biomarker category, Surrogate Biomarkers, were defined as biomarkers that were not directly measured but were inferred using AI/ML applied to other, often less invasive, patient data (**Figure 1B**; Please see **Box 1**. Glossary, for Biomarker category definitions).

Box 1 **Glossary of biomarker types.**

Descriptions of the Susceptibility/Risk, Diagnostic, Prognostic, Predictive, Response, Safety biomarkers were retrieved from the BEST (Biomarkers, EndpointS, and other Tools) Resource (3). BEST defines a biomarker as a defined characteristic that is measured as an indicator of normal biological processes, pathogenic processes, or responses to an exposure or intervention, including therapeutic interventions. Molecular, histologic, radiologic, or physiologic characteristics are types of biomarkers. *Biomarkers that predict histological disease subsets were included under the “Diagnostic Biomarkers” category. Biomarkers that predict molecular or potential molecular subsets were included under the “Surrogate Biomarkers” category.

**Table:** 

Biomarker Type	Description
**Susceptibility/Risk Biomarkers**	A biomarker that indicates the potential for developing a disease or medical condition in an individual who does not currently have clinically apparent disease or the medical condition.
Diagnostic Biomarkers*	A biomarker used to detect or confirm presence of a disease or condition of interest or to identify individuals with a subtype of the disease.
**Prognostic Biomarkers**	A biomarker used to identify likelihood of a clinical event, disease recurrence or progression in patients who have the disease or medical condition of interest.
**Predictive Biomarkers**	A biomarker used to identify individuals who are more likely than similar individuals without the biomarker to experience a favorable or unfavorable effect from exposure to a medical product or an environmental agent.
**Safety Biomarkers**	A biomarker measured before or after an exposure to a medical product or an environmental agent to indicate the likelihood, presence, or extent of toxicity as an adverse effect.
**Response Biomarkers**	A biomarker used to show that a biological response, potentially beneficial or harmful, has occurred in an individual who has been exposed to a medical product or an environmental agent.
**Surrogate Biomarkers**	A biomarker that was not directly measured but was inferred using AI/ML applied to other, often less invasive, patient data.

## Results

### AI/ML-derived susceptibility/risk biomarkers of NSCLC

The risk of developing a complex disease is explained by a combination of genetic and environmental factors. For NSCLC, cigarette smoking is the number one environmental risk factor with smokers being 15-30 times more likely to develop NSCLC than non-smokers (27). Among non-smokers, NSCLC is observed significantly more frequently in females than males, suggesting sex is a risk factor beyond cigarette smoking (28). Exposure to asbestos, radon, or other pollutants have also been reported as environmental risk factors of NSCLC (29). While NSCLC is considered a disease of the elderly with a median patient age of 70 at diagnosis, a subset of NSCLC patients (1-10%) are diagnosed at younger ages (<40 years) (30), indicating potential germline or distinct somatic driver mutations may be present in different patient age groups. Genome-wide association studies focusing on germline genetic variants have reported 16 independent loci associated with risk of developing NSCLC (31) (**Table S1C**). Polygenic risk score models based on the collective effect of these germline genetic variants were shown to successfully predict NSCLC risk beyond age and smoking years (32). While somatic genetic variants are important biomarkers used in selection of targeted therapies, they are not suitable for NSCLC risk assessment, as accessing lung tissue samples cannot be justified for routine risk assessment purposes. Similarly, using non-invasive genetic approaches such as circulating tumor DNA (ctDNA) sequencing is not suitable for risk prediction because ctDNA is at low concentrations even in early-stage cancers (33).

Susceptibility/Risk Biomarkers are defined as biomarkers that indicate the potential for developing a disease or medical condition in an individual who does not currently have clinically apparent disease or the medical condition (3). Using our approach (**Figure 1B**), we found that machine learning studies focused primarily on integrating behavioral risk factors, family history, and environmental factors into NSCLC risk modeling. We found only one study that applied ML to identify biomarkers that could be used for NSCLC risk prediction (34) (**Table S1D**). In this study, Umu et al. reported that ML models of circulating serum RNA levels can predict NSCLC risk 6-8 years before manifestation of disease symptoms and provided evidence that feature selection approaches (i.e., selecting the most discriminative variables while eliminating the redundant or irrelevant variables; please see Pudjihartono et al. (35) for a summary on feature selection algorithms) and histology-specific data subsets may enhance model performance metrics [for model performance metrics including accuracy, recall, specificity, precision, F1-score, please see Hicks et al. (36)].

### AI/ML-derived diagnostic biomarkers of NSCLC

Early symptoms of NSCLC including shortness of breath, fatigue, coughing, and loss of appetite are often mistaken for other conditions due to their non-specific nature. The US Preventive Services Task Force recommends annual risk screening using low-dose CT for high-risk individuals who are between 50 and 80 years old and have at least a 20-pack-year smoking history (37). However, despite these efforts approximately 55% of NSCLC patients present with locally advanced or metastatic disease at the time of diagnosis (38). When NSCLC is suspected, the initial evaluation is performed using imaging tools including chest X-ray, CT, or PET/CT scan. Diagnosis requires histological confirmation using tissue samples stained with Hematoxylin and Eosin (H&E). When tissue morphology is insufficient for proper classification, immunohistochemistry (e.g., TTF-1, Napsin A, CK7, P63, CK5/6) is recommended to aid differential diagnosis (39). While molecular testing of somatic mutations could contribute to diagnosis of NSCLC, current use cases of such testing are primarily limited to informing the treatment plans of already diagnosed patients.

**Figure 2:**
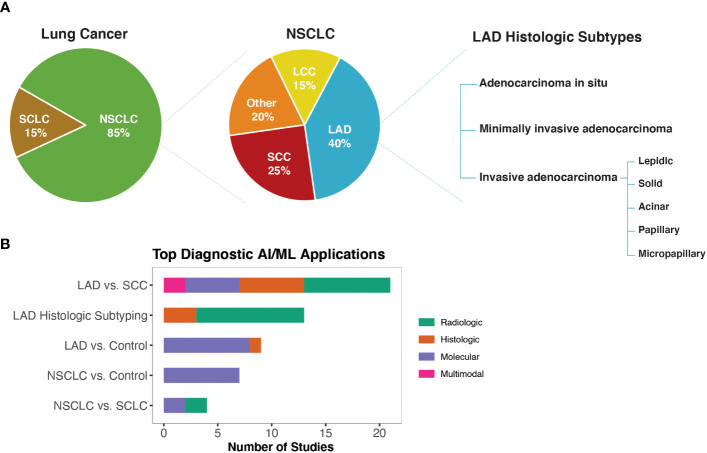
AI/ML Applications for Diagnostic Biomarker Discovery **(A)** Lung cancer, NSCLC, and LAD histologic subtypes, respectively. Subtype frequencies were retrieved from Schabath et al. (40) **(B)** Bar graph of the top five model outcomes/topics where AI/ML algorithms have been developed to identify potential diagnostic biomarkers for NSCLC. Color coding indicates the broad biomarker data type used in these studies (i.e., Molecular, Histologic, Radiologic, Multimodal data types). The acronyms are as follows: SCLC, Small Cell Lung Cancer; NSCLC, Non-Small Cell Lung Cancer; LAD, Lung Adenocarcinoma; SCC, Squamous Cell Carcinoma; LCC, Large Cell Carcinoma.

Diagnostic biomarkers are defined as biomarkers that are used to detect or confirm the presence of a disease or condition of interest or to identify individuals with a subtype of the disease (3) (**Figure 2A**). Using the approach shown in **Figure 1B**, we identified 69 studies that used machine learning approaches to identify potential diagnostic biomarkers of NSCLC (**Table S1E**). Overall, most diagnostic efforts concentrated around building models that could be used to distinguish the two most common histological subtypes of NSCLC; lung adenocarcinoma (LAD) and squamous cell carcinoma (SCC) (41–61). Additionally, several studies have reported AI/ML models and proposed biomarkers that could be used to distinguish NSCLC or LAD from healthy control/non-malignant samples (43, 53, 59, 62–74), as well as for differential diagnosis of NSCLC and SCLC (75–78) (**Figure 2B**).

A body of literature has reported diagnostic AI/ML models leveraging CT or PET/CT radiologic datasets (**Table S1E**). These studies primarily used radiomics or CNN (Convolutional Neural Network)-based approaches to extract image features. Radiomics-based approaches are often criticized for having high variability due to use of manual/semi-automatic tumor segmentation techniques as well as for relying on pre-defined mathematical equations/hand-crafted features. Unlike radiomics-based approaches, CNN-based study designs often build end-to-end algorithms that automate the tissue segmentation, feature extraction, and classifier training steps. Although CNNs offer the potential to reduce human introduced bias, they require larger training datasets compared to radiomics-based approaches and offer less interpretability. In this regard, we identified several studies that integrated radiomics and CNN-based approaches to improve model prediction accuracy while providing clinical interpretability (52, 79–82).

Histology-based diagnosis of NSCLC subtypes can be complex as visual inspections by pathologists are prone to subjective assessments and may result in different interpretations. CNNs trained on H&E-stained Whole Slide Images (WSIs) have shown encouraging results for automated differential diagnosis of LAD vs. SCC as well as for histologic subtyping of LAD growth patterns (60, 83, 84). Despite these efforts, challenges related to the interpretability of CNN-based classifiers as well as computational constraints of high-resolution WSI datasets continue to be obstacles to their widespread clinical utility. Deep feature visualization (i.e., the process of generating visual representations of the features learned from deep neural networks) and resolution-based knowledge distillation (i.e., an approach to transfer knowledge from a high-resolution neural network to a smaller lower-resolution one) were among the emerging approaches to improve interpretability and computational feasibility of deep learning solutions for digital pathology (67, 85).

Molecular biomarkers, in particular somatic driver mutations, are increasingly being used to guide treatment plans for NSCLC patients. Molecular testing of tumor tissue biopsies is currently the gold standard practice to identify actionable molecular biomarkers, but the invasive nature of this process limits its use in routine diagnostic screening. Emerging non-invasive liquid biopsy tests also have limited applications for routine diagnostic screening, as ctDNA is at low concentrations in early-stage cancers (33). An ideal diagnostic biomarker requires low invasiveness and easy detection to allow early diagnosis. However, we found that ML studies that leveraged molecular biomarkers for diagnostic purposes have mainly used genome-wide gene expression data derived from lung tissue. A recurrent finding from these studies was that non-coding RNA expression signatures could differentiate NSCLC/LAD tissue from normal tissue (43, 59, 72, 73). Recapitulating known biology, ML algorithms that used lung gene expression levels to distinguish LAD vs. SCC have reported *TP63*, a known IHC marker for differentiating LAD vs. SCC, as well as several keratin-related genes (e.g., *KRT5*, *KRT6A*, *KRT14*, *SERPINB13*) among the top discriminative features (i.e., top attributes impacting model’s ability to differentiate between different classes) (48, 56). In liquid biopsy-based diagnostic studies, gene-expression signatures from tumor-educated platelets and small extracellular vesicles as well as cfDNA (cell-free DNA) fragmentation patterns (DELFI score; proportion of short (100-150 bp) to long (86–155) cfDNA fragments) were reported as potential biomarkers for NSCLC diagnosis (63, 68, 76).

### AI/ML-derived prognostic biomarkers of NSCLC

**Figure 3:**
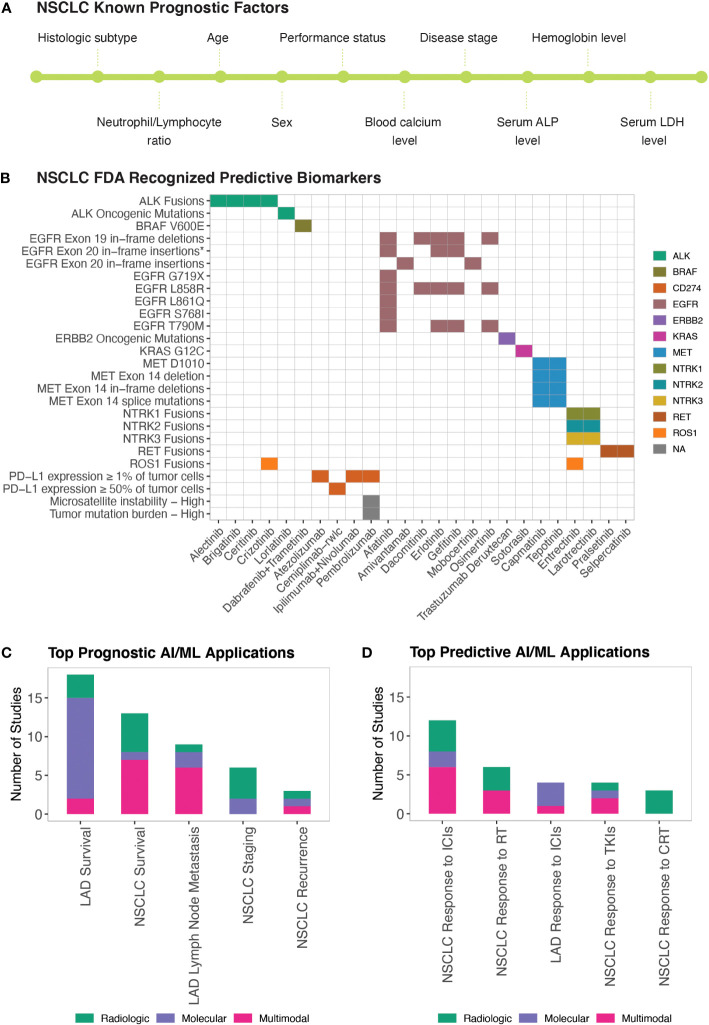
AI/ML Applications for Prognostic and Predictive Biomarker Discovery **(A)** Commonly studied prognostic factors of NSCLC. LDH and ALP stand for Lactate Dehydrogenase and Alkaline Phosphatase, respectively. **(B)** FDA-approved Predictive Biomarkers for Non-Small Cell Lung Cancer. Gene, drug names, and biomarkers were retrieved from the Table of Pharmacogenomic Biomarkers in Drug Labeling (6) in December, 2022. The list of ALK and ERBB2 oncogenic mutations is included in **Table S1A**. *EGFR Exon 20 in-frame insertions (excluding A763_Y764insFQEA) are drug resistance biomarkers for Erlotinib; Gefitinib; Afatinib. **(C)** Bar graph of the top five model outcomes/topics where AI/ML algorithms have been developed to identify potential prognostic biomarkers for NSCLC. Color coding indicates the broad biomarker data type used in these studies (i.e., Molecular, Histologic, Radiologic, Multimodal data types) **(D)** Bar graph of the top five model outcomes/topics where AI/ML algorithms have been developed to identify potential predictive biomarkers for NSCLC. Color coding indicates the broad biomarker data type used in these studies (i.e., Molecular, Histologic, Radiologic, Multimodal data types). ICIs, Immune Checkpoint Inhibitors; TKIs, Tyrosine Kinase Inhibitors; RT, Radiotherapy; CRT, Chemoradiotherapy.

NSCLC prognosis has been correlated with several clinical and demographic parameters including but not limited to the histologic subtype, disease stage, patient performance status, age, sex, blood hemoglobin and calcium levels, blood neutrophil-to-lymphocyte ratio, and serum lactate dehydrogenase and alkaline phosphatase levels (87, 88) (**Figure 3A**). Disease prognosis as well as the therapeutic options for NSCLC also depend on the molecular biology of the tumor (87) (**Figure 3B**). Similarly, Minimal Residual Disease (MRD) (i.e., small number of cancer cells that may remain in the body after cancer treatment and even when patient is in remission) levels have recently started being used in predicting NSCLC relapse risk (89).

Prognostic biomarkers are defined as biomarkers that are used to identify the likelihood of a clinical event, disease recurrence, or progression in patients who have the disease or medical condition of interest (3). Prognostic biomarkers are often confused with predictive biomarkers because predictive biomarkers are associated with prognostic outcomes in response to receiving a particular treatment. With NSCLC having FDA recognized predictive biomarkers (**Table S1A**), cataloguing prognostic biomarkers independent of predictive biomarkers can be misleading because biomarkers that were once associated with unfavorable outcomes can now be associated with favorable outcomes in response to targeted therapies. To identify a predictive biomarker, BEST recommends a comparison of a treatment to a control in patients with and without the biomarker (3). However, upon reviewing AI/ML studies of NSCLC biomarker research, we found that published prognostic and predictive biomarker studies are often confounded in single-arm evaluations. Acknowledging these issues, we used proxy definitions and catalogued studies as “Prognostic” when the prognostic outcomes were investigated regardless of the patients’ treatment status and as “Predictive” when prognostic outcomes were investigated in patient cohorts that were exposed to a specific medical product or an environmental agent.

We identified 58 manuscripts that reported AI/ML models to identify potential prognostic biomarkers of NSCLC (**Table S1F**). The most frequently studied prognostic outcomes were LAD Survival (79, 87, 90–104), NSCLC Survival (105–117), LAD Lymph Node Metastasis (98, 118–124), NSCLC Staging (66, 125–130), and NSCLC Recurrence (131–133) (**Figure 3C**).

Time-to-event is the typical outcome variable when the metric of prognosis is a survival phenotype. However, native ML models cannot handle time-to-event data while accommodating censored observations. Reflecting this, we found that ML studies predicting NSCLC/LAD survival mainly formulated the survival analysis as a classification problem and transformed time-to-event data into dichotomized endpoints (90–94, 96, 100, 102, 103, 106, 108–111, 113, 116, 117, 134, 135). To this end, utilizing Random Survival Forests (RSF) for continuous time-to-event survival prediction and those aiming to identify optimal time-to-event ML models were emerging (98, 99, 101, 105), but further applications and research in this area are warranted.

Prognostic ML studies using CT and PET/CT datasets were primarily based on pre-treatment images (79, 93, 105, 109, 111, 115, 126, 127, 129, 130, 133, 136). These studies emphasized the need for improved multi-institution data integration and image harmonization approaches to help build robust prognostic models (105, 109, 126). Prognostic molecular and multimodal ML studies mainly leveraged tumor gene expression datasets (**Table S1F**). Tumor microenvironment (TME) gene expression signatures have been investigated frequently in the context of developing prognostic ML models for NSCLC (92, 96, 99, 100, 110, 137, 138). In addition to TME immune gene signatures, other components such as hypoxia, pyroptosis, and intercellular communication were prioritized to build prognostic gene models for NSCLC (92, 96, 137).

### AI/ML-derived predictive biomarkers of NSCLC

Clinical response to drugs can be influenced by many factors, including patient age, sex, body mass index, concomitant therapies, genetic make-up, circadian and seasonal variations, and drug absorption, distribution, metabolism, excretion (ADME) profiles. Precision/Personalized Medicine aims to customize treatment regimens based on known variables that predict response to available therapies. Pharmacogenetics and Pharmacogenomics efforts currently are the major driving forces enabling Precision Medicine in NSCLC treatment. Mutations in 11 genes (*EGFR*, *KRAS*, *ALK*, *ROS1*, *BRAF*, *NTRK1*, *NTRK2*, *NTRK3*, *MET*, *RET, ERBB2*), IHC quantified PD-L1 (*CD274*) expression, microsatellite instability, and Tumor Mutation Burden (TMB) constitute the FDA recognized predictive biomarkers predicting response to NSCLC therapies (**Table S1A**; **Figure 3B**). The FDA requires that Companion Diagnostics (CDx) tests/devices are used when screening these predictive biomarkers to accurately identify patient cohorts who are likely to benefit from the therapeutic products.

BEST defines predictive biomarkers as biomarkers that are used to identify individuals who are more likely than similar individuals without the biomarker to experience a favorable or unfavorable effect from exposure to a medical product or an environmental agent (3). In this study, as described in the previous section, we used proxy definitions due to the single arm study designs of the published AI/ML-based prognostic and predictive biomarker studies. We catalogued studies as “Prognostic” when the prognostic outcomes were investigated regardless of the patients’ treatment status and as “Predictive” when prognostic outcomes are investigated in patient cohorts that were exposed to a specific medical product or an environmental agent. We identified 34 manuscripts that used ML approaches to identify potential predictive biomarkers of NSCLC (**Table S1G**). The most frequently studied predictive outcomes were NSCLC/LAD Response to ICIs (96, 100, 139–152), NSCLC Response to Radiotherapy (86, 114, 153–156), NSCLC Response to Tyrosine Kinase Inhibitors (TKIs) (151, 157–159), and NSCLC Response to Chemoradiotherapy (160–162) (**Figure 3D**).

Three tumor-centric biomarkers; PD-L1 expression (≥ 1% or ≥ 50% of tumor cells), Microsatellite Instability (mutations in ≥30% of microsatellites/mismatch repair deficient), and Tumor Mutation Burden (TMB-H; ≥10 somatic mutations/Mb) are FDA-approved biomarkers for ICIs used to treat NSCLC (**Figure 3B**). However, only a fraction of the biomarker-positive NSCLC patients(20-30%) respond to ICI therapies (163). Among the ML studies we compiled, 16 reported predictive biomarkers for ICIs (96, 100, 139–152). In addition to the FDA-approved biomarkers, TMB and PD-L1 tumor proportion score (143, 145), TME-related immune gene signatures (141, 148), neutrophil-to-lymphocyte ratio (142, 143), and mutant allele tumor heterogeneity (MATH) (145) were reported as potential biomarkers predicting response to ICIs.

Mutations in eight genes (*EGFR*, *ALK*, *ROS1*, *NTRK1*, *NTRK2*, *NTRK3*, *MET*, *RET*) are FDA-approved biomarkers predicting response to NSCLC TKIs (**Figure 3B**). For biomarker positive NSCLC patients, the overall response rate to TKIs is more than 60% (164, 165). Through our literature search, we identified four studies that leveraged AI/ML to identify predictive biomarkers for TKIs in NSCLC patients (151, 157–159) and two in LAD-specific cohorts (87, 166). These studies mainly reported radiomics-based predictive models (151, 157, 158, 166). There was a report of a liquid-biopsy based protein signature in patients with *ALK* rearrangements predicting response to crizotinib (159) and an OncoCast ML framework that revealed that mutations in *TP53* and *ARID1A* define a high-risk group with shorter survival in patients who received TKI therapies (87).

Stereotactic body radiation therapy (SBRT) is the standard of care treatment for early-stage NSCLC patients who are not candidates for surgery (153). For qualified patients, local control rate with SBRT treatment is around 90% but, as with surgical patients, distant failure is observed in about 20% of patients (167). Accurate prediction of response to SBRT in NSCLC patients can help identify patients who are more likely to benefit from upfront SBRT vs. systemic therapies. To this end, radiological image-only (114, 154, 155) and multimodal (86, 153, 156) classifiers have been reported, with consistent findings that CNNs demonstrate superior predictive power compared to pre-defined tumor image features (114, 154), and that inclusion of the biologically effective dose (BED) of SBRT improves predictive abilities of the models built (86, 153).

Concurrent chemoradiotherapy (CCRT) is a standard treatment option for stage II and stage III NSCLC patients with unresectable locally advanced cancer. The overall response rate to CCRT is around 80% (168). Identifying patient subsets who may benefit from intensified CCRT is important for better treatment planning. We identified three studies that reported predictive biomarkers for CRT (160–162) (**Figure 3D**). All three studies were based on PET/CT data and here *ad-hoc* consensus and fusion ML approaches were shown to increase the prediction accuracies of the resulting models (161, 162).

### AI/ML-derived safety biomarkers of NSCLC

**Figure 4:**
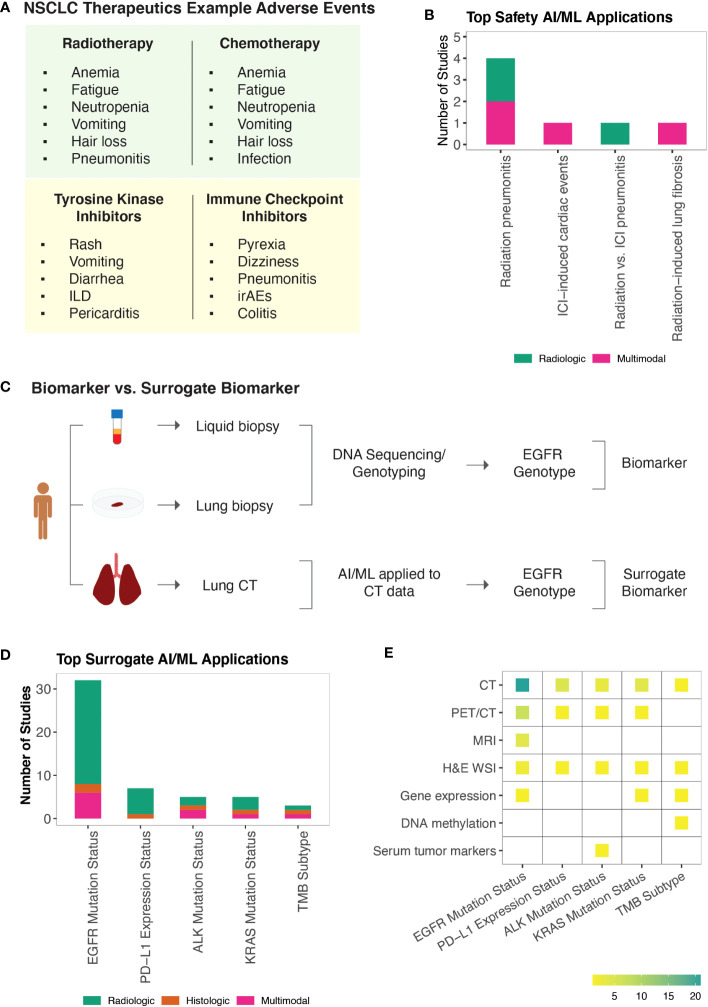
AI/ML Applications for Safety and Surrogate Biomarker Discovery **(A)** NSCLC non-specific and targeted therapies and examples of their known adverse events. Note that adverse events associated with KRAS and Serine/threonine kinase inhibitors are not included. Representative adverse events were pulled from Open Targets Pharmacovigilance tables, which are based on the FDA Adverse Event Reporting database (169, 170) **(B)** Bar graph of the model outcomes/topics where AI/ML algorithms have been developed to identify potential safety biomarkers for NSCLC. Color coding indicates the broad biomarker data type used in these studies (i.e., Molecular, Histologic, Radiologic, Multimodal data types). **(C)** An example illustration of “Biomarker Quantification” (i.e., Direct assay) vs. “Surrogate Biomarker” Prediction (i.e., AI/ML applied to indirect assay data) **(D)** Bar graph of the surrogate biomarker types where biomarker prediction was made through applying AI/ML to other indirect data types. Color coding indicates the broad biomarker data type used in these studies (i.e., Molecular, Histologic, Radiologic, Multimodal data types). **(E)** A heatmap of the indirect biomarker data types that were leveraged through AI/ML applications to predict corresponding surrogate biomarker types. Heatmap density indicates the number of ML studies that used the data types shown on the y-axis to infer NSCLC biomarkers displayed on the x-axis.

The FDA Adverse Event Reporting System (FAERS) is the primary surveillance tool that contains adverse events and safety concerns that are attributed to marketed drugs (169). Early detection of treatment-related adverse events is important for symptom management and successful treatment. Non-specific cancer treatments including radiotherapy and chemotherapy are mainly associated with hematologic (e.g., anemia, neutropenia, fatigue), gastrointestinal (e.g., nausea, vomiting), and dermatological (e.g., skin rashes, hair loss) toxicities. Pneumonitis is among the most severe of toxicities attributed to lung radiotherapy. Targeted therapies have a more variable spectrum of adverse events relative to non-specific cancer treatments (169, 170). TKIs are commonly associated with reversible symptoms including skin changes, vomiting, and diarrhea. However, more serious drug-specific adverse events such as interstitial lung disease and pericarditis have also been reported. Similarly, adverse events from ICIs range from reversible symptoms (e.g., dizziness, pyrexia) to more serious off-target inflammations that are referred to as immune-related adverse events (irAEs) (**Figure 4A**). While mild adverse effects can be managed symptomatically, moderate or severe toxicities necessitate dose reduction or treatment breaks, ultimately impacting drug efficacies. Similarly, many promising combination therapies for NSCLC face challenges due to increased toxicities of drug combinations (171), highlighting the need to identify safety biomarkers that can help tailor treatment approaches or guide the design of clinical trials.

Safety biomarkers are defined as biomarkers measured before or after an exposure to a medical product or an environmental agent to indicate the likelihood, presence, or extent of toxicity as an adverse effect (3). We found seven studies that used AI/ML to identify safety biomarkers that can be used to predict adverse events in response to NSCLC treatments. Potential safety biomarkers were reported for radiation pneumonitis (172–175), radiotherapy-induced lung fibrosis (176), ICI-induced cardiac toxicities (177), and to distinguish radiotherapy-induced vs. ICI-induced pneumonitis in patients who were treated both with radiotherapy and ICIs (178) (**Figure 4B**).

Radiation pneumonitis (RP) is a common (15-40%) complication of lung radiotherapy (179). The severity of RP is tracked using the National Cancer Institute Common Toxicity Criteria with radiation pneumonitis grade ≥ 2 (RP2) being symptomatic and limiting daily living activities. We identified four studies that developed ML models to predict RP2 outcome (172–175) and one study that built a classifier to distinguish radiation vs. ICI induced pneumonitis (178). Here, the concepts of integrating latent and hand-crafted variables (175), dosimetric and radiomic features (173), as well as clinical and baseline cytokine levels (174), were employed to improve the accuracy of RP2 risk prediction models.

Radiation induced lung fibrosis (RILF) is a severe side effect of radiotherapy that significantly impacts quality of life and can lead to non-cancer related death. RILF is classified from grade 0 to grade 5 depending on the clinical manifestation. Accumulating evidence suggests that genetic background as well as cytokines involved in tissue reorganization and immune response modulation are important factors contributing to RILF pathogenesis (180). We identified one ML study that built a classifier to predict RILF risk (176). This study highlighted baseline circulating CCL4 levels, along with dosimetric and clinical parameters as top discriminating features predicting grade ≥ 2 risk (176).

ICI-associated cardiotoxicity is rare but often fatal. Combination immune therapy has been shown to be a risk factor for ICI-associated cardiac events (181). Using our literature search approach, we identified one study that built ML models to predict cardiac events in patients receiving ICI therapy (177). In this study, Heilbroner et al. reported increased age, extremes of weight, presence of cardiac history, low percentage of lymphocytes, and high percentage of neutrophils among the top predictors of ICI-associated cardiotoxicity risk (177).

### AI/ML-derived surrogate biomarkers of NSCLC

Identification of biomarkers from tissue biopsies is challenging due to their invasive nature of collection and small tissue volume, limiting their usefulness for performing repeated measurements, additional tests, and longitudinal monitoring. Non-invasive CDx assays (e.g., cobas EGFR Mutation Test v2, FoundationOne Liquid CDx, Guardant360 CDx) of ctDNA have been approved for certain NSCLC biomarkers (182). Alternative efforts continue to be pursued to detect approved and potential biomarkers of NSCLC. To this end, AI/ML algorithms have been applied to use relatively non-invasive patient data as a substitute to predict clinically approved or other potential biomarker types, which are collectively referred as the surrogate biomarkers in this study (**Figure 4C**).

Under the surrogate biomarker category, we identified 60 ML studies (**Table S1I**). The top predicted biomarkers were *EGFR* mutation status (44, 58, 183–209), PD-L1 expression status (190, 210–215), *ALK* mutation status (94, 216–219), *KRAS* mutation status (44, 194, 197, 220), and TMB subtype (221–223) (**Figures 4D, E**).

In clinical practice, *EGFR* and *KRAS* mutations are routinely detected using DNA-based assays including real-time PCR and sequencing. The detection of PD-L1 expression is based on IHC assays and is considered suboptimal (224). *ALK* mutations or rearrangements can be detected through both DNA- and protein-based assays. There is currently one FDA-approved CDx test for TMB status, which is solid biopsy and sequencing based (182). As a complementary method to existing biomarker tests, we found several ML studies that have demonstrated the value of using CT or PET/CT datasets to predict *EGFR (* 58, 188, 190–195, 197, 198, 200, 201, 203–209), PD-L1 (190, 211–215), *ALK (* 216–219), *KRAS (* 44, 190, 194, 197, 220), and to some extent TMB (223) status. Besides the promise of using radiological image data to predict surrogate molecular biomarkers, the proposed models were also shown to provide potential utility in understanding tumor heterogeneity in which biological inference from different image pixels were shown to reflect intra-tumor heterogeneity (207–209).

In addition to noninvasive radiological data, invasive yet potentially time-efficient and tissue saving alternatives were also reported to be useful in predicting surrogate biomarkers. For example, Sha et al. developed a deep learning model that could predict PD-L1 status from H&E stained WSIs in NSCLC patients (210). Similarly, Chen et al. developed ML models that were trained on H&E stained WSIs to predict multiple genetic aberrations (*ALK*, *BRAF*, *EGFR*, *ROS1* mutation status) and transcriptional subtypes (proximal-inflammatory, proximal-proliferative, terminal respiratory unit) of LAD (94), highlighting the potential of AI/ML approaches to infer different molecular characteristics through the repeated use of the same biological material.

## Discussion

Biomarker discovery is a multifaceted process with many applications in healthcare such as identification of high-risk patients, improving diagnostic accuracies, as well as predicting prognostic outcomes and sensitivity to therapeutics. Despite the advancements in targeted therapies, approximately 30% of NSCLC patients do not harbor known driver mutations, and about 55% do not carry actionable mutations (225, 226). Additionally, even among patients who respond to targeted therapies, adverse events and acquired resistance may interrupt treatment plans, leading to disease progression. Expanding the repertoire of NSCLC biomarkers is critical for both the development of innovative treatments as well as for monitoring disease progression and adverse events. Here, to our knowledge, we report the first comprehensive review of AI/ML applications in the NSCLC biomarker space, catalogue the clinical challenges that are targeted by these studies, and summarize emerging patterns that could inform researchers and clinicians in this field.

Formally, ML is a sub-field of AI and the approaches used across the 215 manuscripts we catalogued could have fit under the ML category, however we used AI/ML interchangeably as this was the case in most published manuscripts. Similarly, the difference between ML and traditional statistics has been the subject of many controversies (227). ML can be built upon both statistical and algorithmic frameworks and common statistical methods can be used for both inferential and predictive modeling. We therefore relied on authors’ self-declaration regarding the use of AI/ML methods in predicting potential biomarkers for NSCLC. Additionally, starting in 2014, guidelines such as CHARMS (22), MLP-BIOM (23), TRIPOD (24), and PROBAST (25) have been published to improve the reporting and appraisal of the prediction models used for diagnostic and prognostic purposes. However, we did not evaluate the manuscripts based on these checklists as our goal was to catalogue the ongoing AI/ML efforts in NSCLC biomarker research rather than assessing the immediate clinical utility of the proposed models or biomarkers.

We catalogued 215 studies identified with respect to the BEST (3) biomarker sub-types (**Figure 1B**). We did not find any AI/ML-derived biomarkers that could fit under the Response Biomarker category (i.e., a biomarker used to show that a biological response, potentially beneficial or harmful, has occurred in an individual who has been exposed to a medical product or an environmental agent). We instead included a new biomarker category, which we referred to as Surrogate Biomarkers, where AI/ML algorithms have been applied to relatively non-invasive patient data to predict the presence of clinically approved or other potential biomarkers of NSCLC. While biomarker discovery is often formulated as a feature selection problem (228), we also included studies that did not select features but had reported classification utility with respect to relevant organismal phenotypes under each biomarker category.

As expected, the models proposed have not been evaluated for their clinical utility. However, the clinical questions, computational challenges, and emerging solutions discussed here can serve as a reference for clinicians and data scientists leveraging biomarker datasets and AI/ML in medicine. Transfer learning methods to relax training set requirements, data harmonization algorithms to minimize technical variability in data generation, the contexts of feature selection and stability to allow interpretable models are among areas that are rapidly advancing. In addition to the stochastic nature of AI/ML models, tumor-specific temporal and spatial molecular heterogeneities, the dynamic composition of the TME, and limitations in tumor tissue access continue to further challenge the evolving landscape of biomarker modeling for NSCLC. Non-invasive approaches including liquid biopsy-based biomarkers and surrogate biomarkers inferred through the use of AI/ML, hold promise to navigate these limitations and advance our understanding of the dynamic nature of tumor progression. Longitudinal data generated through non-invasive means can, however, pose a new challenge; the data generated can be overwhelmingly large as well as complex to analyze and interpret efficiently using traditional methods. The use of automated AI/ML tools in clinical monitoring may thus be essential to facilitate efficient analysis of the substantial amounts of longitudinal biomarker data.

Of note, while AI/ML models have been used (229) and show potential for numerous applications in clinical trials, including opportunities to enhance trial design, safety monitoring, and predictive analytics, there are currently no FDA-released guidelines or performance metrics specific to the use or evaluation of AI/ML algorithms in clinical trials (230). To this end, we anticipate that FDA guidelines for regulating AI/ML-based medical devices (231) and CONSORT-AI (232) recommendations for reporting AI-interventions in trials will facilitate the development of a formalized regulatory process, enabling effective and robust use of AI/ML in clinical trials.

Identification, cataloguing, and continuous updating of emerging biomarkers can expedite the clinical adoption of the innovative biomarkers and technologies. Here, we provided an overview of the fast-growing AI/ML applications in NSCLC biomarker discovery space and discussed the gaps and challenges in the field. By compiling relevant literature on NSCLC biomarker discovery, we revealed a comprehensive picture of the clinical challenges that are commonly targeted using AI/ML approaches and highlighted potential biomarkers and signatures that once adequately appraised may be translated into clinical decision support systems.

## Author contributions

MÇ: Conceptualization, Data curation, Investigation, Methodology, Visualization, Writing – original draft, Formal analysis. KT: Methodology, Validation, Writing – review & editing, Investigation.

## References

[1] Duma N Santana-Davila R Molina JR. Non-small cell lung cancer: epidemiology, screening, diagnosis, and treatment. Mayo Clin Proc (2019) 94(8):1623–40. doi: 10.1016/j.mayocp.2019.01.013 31378236

[2] Siegel RL Miller KD Fuchs HE Jemal A. Cancer statistics, 2022. CA Cancer J Clin (2022) 72(1):7–33. doi: 10.3322/caac.21708 35020204

[3] FDA-NIH Biomarker Working Group. BEST (Biomarkers, EndpointS, and other Tools) Resource. Silver Spring (MD) Bethesda (MD (2016). 27010052

[4] Srivastava S Wagner PD. The early detection research network: A national infrastructure to support the discovery, development, and validation of cancer biomarkers. Cancer Epidemiol Biomarkers Prev (2020) 29(12):2401–10. doi: 10.1158/1055-9965.EPI-20-0237 PMC11106613 32357955

[5] Whirl-Carrillo M McDonagh EM Hebert JM Gong L Sangkuhl K Thorn CF. Pharmacogenomics knowledge for personalized medicine. Clin Pharmacol Ther (2012) 92(4):414–7. doi: 10.1038/clpt.2012.96 PMC3660037 22992668

[6] US Food and Drug Administration Table of Pharmacogenomic Biomarkers in Drug Labeling Available at: https://www.fda.gov/drugs/science-and-research-drugs/table-pharmacogenomic-biomarkers-drug-labeling.

[7] Chakravarty D Gao J Phillips SM Kundra R Zhang H Wang J. OncoKB: A precision oncology knowledge base. JCO Precis Oncol (2017) 2017. doi: 10.1200/PO.17.00011 PMC5586540 28890946

[8] Tate JG Bamford S Jubb HC Sondka Z Beare DM Bindal N. COSMIC: the catalogue of somatic mutations in cancer. Nucleic Acids Res (2019) 47(D1):D941–D7. doi: 10.1093/nar/gky1015 PMC6323903 30371878

[9] Landrum MJ Chitipiralla S Brown GR Chen C Gu B Hart J. ClinVar: improvements to accessing data. Nucleic Acids Res (2020) 48(D1):D835–D44. doi: 10.1093/nar/gkz972 PMC6943040 31777943

[10] Zhang J Bajari R Andric D Gerthoffert F Lepsa A Nahal-Bose H. The international cancer genome consortium data portal. Nat Biotechnol (2019) 37(4):367–9. doi: 10.1038/s41587-019-0055-9 30877282

[11] Jain N Mittendorf KF Holt M Lenoue-Newton M Maurer I Miller C. The My Cancer Genome clinical trial data model and trial curation workflow. J Am Med Inform Assoc (2020) 27(7):1057–66. doi: 10.1093/jamia/ocaa066 PMC7647323 32483629

[12] Zwanenburg A Vallieres M Abdalah MA Aerts H Andrearczyk V Apte A. The image biomarker standardization initiative: standardized quantitative radiomics for high-throughput image-based phenotyping. Radiology. (2020) 295(2):328–38. doi: 10.1148/radiol.2020191145 PMC7193906 32154773

[13] Prior FW Clark K Commean P Freymann J Jaffe C Kirby J. TCIA: An information resource to enable open science. Annu Int Conf IEEE Eng Med Biol Soc (2013) 2013:1282–5. doi: 10.1109/EMBC.2013.6609742 PMC4257783 24109929

[14] Benjamin DJ Haslam A Gill J Prasad V. Targeted therapy in lung cancer: Are we closing the gap in years of life lost? Cancer Med (2022) 11(18):3417–24. doi: 10.1002/cam4.4703 PMC9487872 35315222

[15] Siegel RL Miller KD Jemal A. Cancer statistics, 2018. CA Cancer J Clin (2018) 68(1):7–30. doi: 10.3322/caac.21442 29313949

[16] Nagasaka M Singh V Baca Y Sukari A Kim C Mamdani H. The effects of HER2 alterations in EGFR mutant non-small cell lung cancer. Clin Lung Cancer. (2022) 23(1):52–9. doi: 10.1016/j.cllc.2021.08.012 34801409

[17] Passaro A Attili I Rappa A Vacirca D Ranghiero A Fumagalli C. Genomic characterization of concurrent alterations in non-small cell lung cancer (NSCLC) harboring actionable mutations. Cancers (Basel) (2021) 13(9). doi: 10.3390/cancers13092172 PMC8124171 33946519

[18] Zhao Y Wang S Yang Z Dong Y Wang Y Zhang L. Co-occurring potentially actionable oncogenic drivers in non-small cell lung cancer. Front Oncol (2021) 11:665484. doi: 10.3389/fonc.2021.665484 34221980 PMC8242190

[19] Artificial Intelligence and Machine Learning (AI/ML)-Enabled Medical Devices Available at: https://www.fda.gov/medical-devices/software-medical-device-samd/artificial-intelligence-and-machine-learning-aiml-enabled-medical-devices?utmsource=FDALinkedin#resources.

[20] Senaras C Niazi MKK Lozanski G Gurcan MN. DeepFocus: Detection of out-of-focus regions in whole slide digital images using deep learning. PloS One (2018) 13(10):e0205387. doi: 10.1371/journal.pone.0205387 30359393 PMC6201886

[21] Wang S Wang T Yang L Yang DM Fujimoto J Yi F. ConvPath: A software tool for lung adenocarcinoma digital pathological image analysis aided by a convolutional neural network. EBioMedicine. (2019) 50:103–10. doi: 10.1016/j.ebiom.2019.10.033 PMC6921240 31767541

[22] Moons KG de Groot JA Bouwmeester W Vergouwe Y Mallett S Altman DG. Critical appraisal and data extraction for systematic reviews of prediction modelling studies: the CHARMS checklist. PloS Med (2014) 11(10):e1001744. doi: 10.1371/journal.pmed.1001744 25314315 PMC4196729

[23] Luo W Phung D Tran T Gupta S Rana S Karmakar C. Guidelines for developing and reporting machine learning predictive models in biomedical research: A multidisciplinary view. J Med Internet Res (2016) 18(12):e323. doi: 10.2196/jmir.5870 27986644 PMC5238707

[24] Collins GS Reitsma JB Altman DG Moons KG. Transparent reporting of a multivariable prediction model for individual prognosis or diagnosis (TRIPOD): the TRIPOD statement. BMJ (2015) 350:g7594. doi: 10.1161/CIRCULATIONAHA.114.014508 25569120

[25] Wolff RF Moons KGM Riley RD Whiting PF Westwood M Collins GS. PROBAST: A tool to assess the risk of bias and applicability of prediction model studies. Ann Intern Med (2019) 170(1):51–8. doi: 10.7326/M18-1376 30596875

[26] Malone J Holloway E Adamusiak T Kapushesky M Zheng J Kolesnikov N. Modeling sample variables with an Experimental Factor Ontology. Bioinformatics. (2010) 26(8):1112–8. doi: 10.1093/bioinformatics/btq099 PMC2853691 20200009

[27] Shane B Clark SA. Non small cell lung cancer. (2022).

[28] Baiu I Titan AL Martin LW Wolf A Backhus L. The role of gender in non-small cell lung cancer: a narrative review. J Thorac Dis (2021) 13(6):3816–26. doi: 10.21037/jtd-20-3128 PMC8264700 34277072

[29] Zappa C Mousa SA. Non-small cell lung cancer: current treatment and future advances. Transl Lung Cancer Res (2016) 5(3):288–300. doi: 10.21037/tlcr.2016.06.07 27413711 PMC4931124

[30] Thomas A Chen Y Yu T Jakopovic M Giaccone G. Trends and characteristics of young non-small cell lung cancer patients in the United States. Front Oncol (2015) 5:113. doi: 10.3389/fonc.2015.00113 26075181 PMC4443720

[31] Buniello A MacArthur JAL Cerezo M Harris LW Hayhurst J Malangone C. The NHGRI-EBI GWAS Catalog of published genome-wide association studies, targeted arrays and summary statistics 2019. Nucleic Acids Res (2019) 47(D1):D1005–D12. doi: 10.1093/nar/gky1120 PMC6323933 30445434

[32] Dai J Lv J Zhu M Wang Y Qin N Ma H. Identification of risk loci and a polygenic risk score for lung cancer: a large-scale prospective cohort study in Chinese populations. Lancet Respir Med (2019) 7(10):881–91. doi: 10.1016/S2213-2600(19)30144-4 PMC7015703 31326317

[33] Gao Q Zeng Q Wang Z Li C Xu Y Cui P. Circulating cell-free DNA for cancer early detection. Innovation (Camb). (2022) 3(4):100259. doi: 10.1016/j.xinn.2022.100259 35647572 PMC9133648

[34] Umu SU Langseth H Zuber V Helland A Lyle R Rounge TB. Serum RNAs can predict lung cancer up to 10 years prior to diagnosis. Elife. (2022) 11. doi: 10.7554/eLife.71035 PMC8884722 35147498

[35] Pudjihartono N Fadason T Kempa-Liehr AW O'Sullivan JM. A review of feature selection methods for machine learning-based disease risk prediction. Front Bioinform (2022) 2:927312. doi: 10.3389/fbinf.2022.927312 36304293 PMC9580915

[36] Hicks SA Strumke I Thambawita V Hammou M Riegler MA Halvorsen P. On evaluation metrics for medical applications of artificial intelligence. Sci Rep (2022) 12(1):5979. doi: 10.1038/s41598-022-09954-8 35395867 PMC8993826

[37] Force USPST Krist AH Davidson KW Mangione CM Barry MJ Cabana M. Screening for lung cancer: US preventive services task force recommendation statement. JAMA. (2021) 325(10):962–70. doi: 10.1001/jama.2021.1117 33687470

[38] Zigman Suchsland M Kowalski L Burkhardt HA Prado MG Kessler LG Yetisgen M. How timely is diagnosis of lung cancer? Cohort study of individuals with lung cancer presenting in ambulatory care in the United States. Cancers (Basel) (2022) 14(23). doi: 10.3390/cancers14235756 PMC9740627 36497238

[39] Gurda GT Zhang L Wang Y Chen L Geddes S Cho WC. Utility of five commonly used immunohistochemical markers TTF-1, Napsin A, CK7, CK5/6 and P63 in primary and metastatic adenocarcinoma and squamous cell carcinoma of the lung: a retrospective study of 246 fine needle aspiration cases. Clin Transl Med (2015) 4:16. doi: 10.1186/s40169-015-0057-2 25977750 PMC4417108

[40] Schabath MB Cote ML. Cancer progress and priorities: lung cancer. Cancer Epidemiol Biomarkers Prev (2019) 28(10):1563–79. doi: 10.1158/1055-9965.EPI-19-0221 PMC6777859 31575553

[41] Jain D Nambirajan A Chen G Geisinger K Hiroshima K Layfield L. NSCLC subtyping in conventional cytology: results of the international association for the study of lung cancer cytology working group survey to determine specific cytomorphologic criteria for adenocarcinoma and squamous cell carcinoma. J Thorac Oncol (2022) 17(6):793–805. doi: 10.1016/j.jtho.2022.02.013 35331963

[42] Janssen C Boskamp T Hauberg-Lotte L Behrmann J Deininger SO Kriegsmann M. Robust subtyping of non-small cell lung cancer whole sections through MALDI mass spectrometry imaging. Proteomics Clin Appl (2022) 16(4):e2100068. doi: 10.1002/prca.202100068 35238465

[43] Sulewska A Niklinski J Charkiewicz R Karabowicz P Biecek P Baniecki H. A signature of 14 long non-coding RNAs (lncRNAs) as a step towards precision diagnosis for NSCLC. Cancers (Basel) (2022) 14(2). doi: 10.3390/cancers14020439 PMC8773641 35053601

[44] Trivizakis E Souglakos J Karantanas A Marias K. Deep radiotranscriptomics of non-small cell lung carcinoma for assessing molecular and histology subtypes with a data-driven analysis. Diagnostics (Basel). (2021) 11(12). doi: 10.3390/diagnostics11122383 PMC8700168 34943617

[45] Le Page AL Ballot E Truntzer C Derangere V Ilie A Rageot D. Using a convolutional neural network for classification of squamous and non-squamous non-small cell lung cancer based on diagnostic histopathology HES images. Sci Rep (2021) 11(1):23912. doi: 10.21203/rs.3.rs-646715/v1 34903781 PMC8669012

[46] Neumann JM Freitag H Hartmann JS Niehaus K Galanis M Griesshammer M. Subtyping non-small cell lung cancer by histology-guided spatial metabolomics. J Cancer Res Clin Oncol (2022) 148(2):351–60. doi: 10.1007/s00432-021-03834-w PMC8800912 34839410

[47] Dehkharghanian T Rahnamayan S Riasatian A Bidgoli AA Kalra S Zaveri M. Selection, visualization, and interpretation of deep features in lung adenocarcinoma and squamous cell carcinoma. Am J Pathol (2021) 191(12):2172–83. doi: 10.1016/j.ajpath.2021.08.013 34508689

[48] Chen JW Dhahbi J. Lung adenocarcinoma and lung squamous cell carcinoma cancer classification, biomarker identification, and gene expression analysis using overlapping feature selection methods. Sci Rep (2021) 11(1):13323. doi: 10.1038/s41598-021-92725-8 34172784 PMC8233431

[49] Liu H Jiao Z Han W Jing B. Identifying the histologic subtypes of non-small cell lung cancer with computed tomography imaging: a comparative study of capsule net, convolutional neural network, and radiomics. Quant Imaging Med Surg (2021) 11(6):2756–65. doi: 10.21037/qims-20-734 PMC8107316 34079739

[50] Aydin N Celik O Aslan AF Odabas A Dundar E Sahin MC. Detection of lung cancer on computed tomography using artificial intelligence applications developed by deep learning methods and the contribution of deep learning to the classification of lung carcinoma. Curr Med Imaging. (2021) 17(9):1137–41. doi: 10.2174/1573405617666210204210500 33563200

[51] Yang F Chen W Wei H Zhang X Yuan S Qiao X. Machine learning for histologic subtype classification of non-small cell lung cancer: A retrospective multicenter radiomics study. Front Oncol (2020) 10:608598. doi: 10.3389/fonc.2020.608598 33520719 PMC7840845

[52] Marentakis P Karaiskos P Kouloulias V Kelekis N Argentos S Oikonomopoulos N. Lung cancer histology classification from CT images based on radiomics and deep learning models. Med Biol Eng Comput (2021) 59(1):215–26. doi: 10.1007/s11517-020-02302-w 33411267

[53] Wang J Xie X Shi J He W Chen Q Chen L. Denoising autoencoder, A deep learning algorithm, aids the identification of A novel molecular signature of lung adenocarcinoma. Genomics Proteomics Bioinf (2020) 18(4):468–80. doi: 10.1016/j.gpb.2019.02.003 PMC8242334 33346087

[54] Ren C Zhang J Qi M Zhang J Zhang Y Song S. Machine learning based on clinico-biological features integrated (18)F-FDG PET/CT radiomics for distinguishing squamous cell carcinoma from adenocarcinoma of lung. Eur J Nucl Med Mol Imaging. (2021) 48(5):1538–49. doi: 10.1007/s00259-020-05065-6 PMC8113203 33057772

[55] Han Y Ma Y Wu Z Zhang F Zheng D Liu X. Histologic subtype classification of non-small cell lung cancer using PET/CT images. Eur J Nucl Med Mol Imaging. (2021) 48(2):350–60. doi: 10.1007/s00259-020-04771-5 32776232

[56] Yuan F Lu L Zou Q. Analysis of gene expression profiles of lung cancer subtypes with machine learning algorithms. Biochim Biophys Acta Mol Basis Dis (2020) 1866(8):165822. doi: 10.1016/j.bbadis.2020.165822 32360590

[57] Hyun SH Ahn MS Koh YW Lee SJ. A machine-learning approach using PET-based radiomics to predict the histological subtypes of lung cancer. Clin Nucl Med (2019) 44(12):956–60. doi: 10.1097/RLU.0000000000002810 31689276

[58] Koyasu S Nishio M Isoda H Nakamoto Y Togashi K. Usefulness of gradient tree boosting for predicting histological subtype and EGFR mutation status of non-small cell lung cancer on (18)F FDG-PET/CT. Ann Nucl Med (2020) 34(1):49–57. doi: 10.1007/s12149-019-01414-0 31659591

[59] Sherafatian M Arjmand F. Decision tree-based classifiers for lung cancer diagnosis and subtyping using TCGA miRNA expression data. Oncol Lett (2019) 18(2):2125–31. doi: 10.3892/ol.2019.10462 PMC6607095 31423286

[60] Coudray N Ocampo PS Sakellaropoulos T Narula N Snuderl M Fenyo D. Classification and mutation prediction from non-small cell lung cancer histopathology images using deep learning. Nat Med (2018) 24(10):1559–67. doi: 10.1038/s41591-018-0177-5 PMC9847512 30224757

[61] Zhu X Dong D Chen Z Fang M Zhang L Song J. Radiomic signature as a diagnostic factor for histologic subtype classification of non-small cell lung cancer. Eur Radiol (2018) 28(7):2772–8. doi: 10.1007/s00330-017-5221-1 29450713

[62] Wang S Wang Q Fan B Gong J Sun L Hu B. Machine learning-based screening of the diagnostic genes and their relationship with immune-cell infiltration in patients with lung adenocarcinoma. J Thorac Dis (2022) 14(3):699–711. doi: 10.21037/jtd-22-206 35399247 PMC8987822

[63] Li W Zhu L Li K Ye S Wang H Wang Y. Machine learning-assisted dual-marker detection in serum small extracellular vesicles for the diagnosis and prognosis prediction of non-small cell lung cancer. Nanomaterials (Basel) (2022) 12(5). doi: 10.3390/nano12050809 PMC8912499 35269297

[64] Li C Tian C Zeng Y Liang J Yang Q Gu F. Integrated analysis of MATH-based subtypes reveals a novel screening strategy for early-stage lung adenocarcinoma. Front Cell Dev Biol (2022) 10:769711. doi: 10.3389/fcell.2022.769711 35211471 PMC8861524

[65] Pedraz-Valdunciel C Giannoukakos S Potie N Gimenez-Capitan A Huang CY Hackenberg M. Digital multiplexed analysis of circular RNAs in FFPE and fresh non-small cell lung cancer specimens. Mol Oncol (2022) 16(12):2367–83. doi: 10.1002/1878-0261.13182 PMC9208080 35060299

[66] Lu H Gao NL Tong F Wang J Li H Zhang R. Alterations of the human lung and gut microbiomes in non-small cell lung carcinomas and distant metastasis. Microbiol Spectr. (2021) 9(3):e0080221. doi: 10.1128/Spectrum.00802-21 34787462 PMC8597645

[67] Lin CK Chang J Huang CC Wen YF Ho CC Cheng YC. Effectiveness of convolutional neural networks in the interpretation of pulmonary cytologic images in endobronchial ultrasound procedures. Cancer Med (2021) 10(24):9047–57. doi: 10.1002/cam4.4383 PMC8683546 34725953

[68] Goswami C Chawla S Thakral D Pant H Verma P Malik PS. Molecular signature comprising 11 platelet-genes enables accurate blood-based diagnosis of NSCLC. BMC Genomics (2020) 21(1):744. doi: 10.1186/s12864-020-07147-z 33287695 PMC7590669

[69] Huang L Wang L Hu X Chen S Tao Y Su H. Machine learning of serum metabolic patterns encodes early-stage lung adenocarcinoma. Nat Commun (2020) 11(1):3556. doi: 10.1038/s41467-019-14242-7 32678093 PMC7366718

[70] Noreldeen HAA Du L Li W Liu X Wang Y Xu G. Serum lipidomic biomarkers for non-small cell lung cancer in nonsmoking female patients. J Pharm BioMed Anal (2020) 185:113220. doi: 10.1016/j.jpba.2020.113220 32145537

[71] Shen N Du J Zhou H Chen N Pan Y Hoheisel JD. A diagnostic panel of DNA methylation biomarkers for lung adenocarcinoma. Front Oncol (2019) 9:1281. doi: 10.3389/fonc.2019.01281 31850197 PMC6901798

[72] Smolander J Stupnikov A Glazko G Dehmer M Emmert-Streib F. Comparing biological information contained in mRNA and non-coding RNAs for classification of lung cancer patients. BMC Cancer. (2019) 19(1):1176. doi: 10.1186/s12885-019-6338-1 31796020 PMC6892207

[73] Wang Y Fu J Wang Z Lv Z Fan Z Lei T. Screening key lncRNAs for human lung adenocarcinoma based on machine learning and weighted gene co-expression network analysis. Cancer biomark (2019) 25(4):313–24. doi: 10.3233/CBM-190225 PMC12828841 31322548

[74] Xiao Y Wu J Lin Z Zhao X. A deep learning-based multi-model ensemble method for cancer prediction. Comput Methods Programs Biomed (2018) 153:1–9. doi: 10.1016/j.cmpb.2017.09.005 29157442

[75] Xie Z Zhang H. Analysis of the diagnosis model of peripheral non-small-cell lung cancer under computed tomography images. J Healthc Eng. (2022) 2022:3107965. doi: 10.1155/2022/3107965 35222880 PMC8881128

[76] Mathios D Johansen JS Cristiano S Medina JE Phallen J Larsen KR. Detection and characterization of lung cancer using cell-free DNA fragmentomes. Nat Commun (2021) 12(1):5060. doi: 10.1038/s41467-021-24994-w 34417454 PMC8379179

[77] Chen BT Chen Z Ye N Mambetsariev I Fricke J Daniel E. Differentiating peripherally-located small cell lung cancer from non-small cell lung cancer using a CT radiomic approach. Front Oncol (2020) 10:593. doi: 10.3389/fonc.2020.00593 32391274 PMC7188953

[78] O'Shea K Cameron SJ Lewis KE Lu C Mur LA. Metabolomic-based biomarker discovery for non-invasive lung cancer screening: A case study. Biochim Biophys Acta (2016) 1860(11 Pt B):2682–7. doi: 10.1016/j.bbagen.2016.07.007 27423423

[79] Wang C Shao J Lv J Cao Y Zhu C Li J. Deep learning for predicting subtype classification and survival of lung adenocarcinoma on computed tomography. Transl Oncol (2021) 14(8):101141. doi: 10.1016/j.tranon.2021.101141 34087705 PMC8184655

[80] Wang X Li Q Cai J Wang W Xu P Zhang Y. Predicting the invasiveness of lung adenocarcinomas appearing as ground-glass nodule on CT scan using multi-task learning and deep radiomics. Transl Lung Cancer Res (2020) 9(4):1397–406. doi: 10.21037/tlcr-20-370 PMC7481614 32953512

[81] Xia X Gong J Hao W Yang T Lin Y Wang S. Comparison and fusion of deep learning and radiomics features of ground-glass nodules to predict the invasiveness risk of stage-I lung adenocarcinomas in CT scan. Front Oncol (2020) 10:418. doi: 10.3389/fonc.2020.00418 32296645 PMC7136522

[82] Lu L Wang D Wang L E L Guo P Li Z. A quantitative imaging biomarker for predicting disease-free-survival-associated histologic subgroups in lung adenocarcinoma. Eur Radiol (2020) 30(7):3614–23. doi: 10.1007/s00330-020-06663-6 PMC9039366 32086583

[83] Wei JW Tafe LJ Linnik YA Vaickus LJ Tomita N Hassanpour S. Pathologist-level classification of histologic patterns on resected lung adenocarcinoma slides with deep neural networks. Sci Rep (2019) 9(1):3358. doi: 10.1038/s41598-019-40041-7 30833650 PMC6399447

[84] Gertych A Swiderska-Chadaj Z Ma Z Ing N Markiewicz T Cierniak S. Convolutional neural networks can accurately distinguish four histologic growth patterns of lung adenocarcinoma in digital slides. Sci Rep (2019) 9(1):1483. doi: 10.1038/s41598-018-37638-9 30728398 PMC6365499

[85] DiPalma J Suriawinata AA Tafe LJ Torresani L Hassanpour S. Resolution-based distillation for efficient histology image classification. Artif Intell Med (2021) 119:102136. doi: 10.1016/j.artmed.2021.102136 34531005 PMC8449014

[86] Hindocha S Charlton TG Linton-Reid K Hunter B Chan C Ahmed M. A comparison of machine learning methods for predicting recurrence and death after curative-intent radiotherapy for non-small cell lung cancer: Development and validation of multivariable clinical prediction models. EBioMedicine. (2022) 77:103911. doi: 10.1016/j.ebiom.2022.103911 35248997 PMC8897583

[87] Shen R Martin A Ni A Hellmann M Arbour KC Jordan E. Harnessing clinical sequencing data for survival stratification of patients with metastatic lung adenocarcinomas. JCO Precis Oncol (2019) 3. doi: 10.1200/PO.18.00307 PMC6474404 31008437

[88] Shimizu K Okita R Saisho S Maeda A Nojima Y Nakata M. Preoperative neutrophil/lymphocyte ratio and prognostic nutritional index predict survival in patients with non-small cell lung cancer. World J Surg Oncol (2015) 13:291. doi: 10.1186/s12957-015-0710-7 26424708 PMC4590710

[89] Frisone D Friedlaender A Addeo A. The role and impact of minimal residual disease in NSCLC. Curr Oncol Rep (2021) 23(12):136. doi: 10.1007/s11912-021-01131-w 34735646 PMC8568856

[90] Dessie EY Chang JG Chang YS. A nine-gene signature identification and prognostic risk prediction for patients with lung adenocarcinoma using novel machine learning approach. Comput Biol Med (2022) 145:105493. doi: 10.1016/j.compbiomed.2022.105493 35447457

[91] Liu Y Yang M Sun W Zhang M Sun J Wang W. Developing prognostic gene panel of survival time in lung adenocarcinoma patients using machine learning. Transl Cancer Res (2020) 9(6):3860–9. doi: 10.21037/tcr-19-2739 PMC8799101 35117753

[92] Liu LP Lu L Zhao QQ Kou QJ Jiang ZZ Gui R. Identification and validation of the pyroptosis-related molecular subtypes of lung adenocarcinoma by bioinformatics and machine learning. Front Cell Dev Biol (2021) 9:756340. doi: 10.3389/fcell.2021.756340 34805165 PMC8599430

[93] Cho HH Lee HY Kim E Lee G Kim J Kwon J. Radiomics-guided deep neural networks stratify lung adenocarcinoma prognosis from CT scans. Commun Biol (2021) 4(1):1286. doi: 10.1038/s42003-021-02814-7 34773070 PMC8590002

[94] Chen L Zeng H Xiang Y Huang Y Luo Y Ma X. Histopathological images and multi-omics integration predict molecular characteristics and survival in lung adenocarcinoma. Front Cell Dev Biol (2021) 9:720110. doi: 10.3389/fcell.2021.720110 34708036 PMC8542778

[95] Min KW Kim DH Noh YK Son BK Kwon MJ Moon JY. Cancer-associated fibroblasts are associated with poor prognosis in solid type of lung adenocarcinoma in a machine learning analysis. Sci Rep (2021) 11(1):16779. doi: 10.1038/s41598-021-96344-1 34408230 PMC8373913

[96] Shi R Bao X Unger K Sun J Lu S Manapov F. Identification and validation of hypoxia-derived gene signatures to predict clinical outcomes and therapeutic responses in stage I lung adenocarcinoma patients. Theranostics. (2021) 11(10):5061–76. doi: 10.7150/thno.56202 PMC7978303 33754044

[97] Deng F Shen L Wang H Zhang L. Classify multicategory outcome in patients with lung adenocarcinoma using clinical, transcriptomic and clinico-transcriptomic data: machine learning versus multinomial models. Am J Cancer Res (2020) 10(12):4624–39. PMC7783755 33415023

[98] Cai Q He B Zhang P Zhao Z Peng X Zhang Y. Exploration of predictive and prognostic alternative splicing signatures in lung adenocarcinoma using machine learning methods. J Transl Med (2020) 18(1):463. doi: 10.1186/s12967-020-02635-y 33287830 PMC7720605

[99] Xue L Bi G Zhan C Zhang Y Yuan Y Fan H. Development and validation of a 12-gene immune relevant prognostic signature for lung adenocarcinoma through machine learning strategies. Front Oncol (2020) 10:835. doi: 10.3389/fonc.2020.00835 32537435 PMC7267039

[100] Bao X Shi R Zhao T Wang Y. Mast cell-based molecular subtypes and signature associated with clinical outcome in early-stage lung adenocarcinoma. Mol Oncol (2020) 14(5):917–32. doi: 10.1002/1878-0261.12670 PMC7191192 32175651

[101] Ma B Geng Y Meng F Yan G Song F. Identification of a sixteen-gene prognostic biomarker for lung adenocarcinoma using a machine learning method. J Cancer. (2020) 11(5):1288–98. doi: 10.7150/jca.34585 PMC6959071 31956375

[102] Li Y Ge D Gu J Xu F Zhu Q Lu C. A large cohort study identifying a novel prognosis prediction model for lung adenocarcinoma through machine learning strategies. BMC Cancer. (2019) 19(1):886. doi: 10.1186/s12885-019-6101-7 31488089 PMC6729062

[103] Shin B Park S Hong JH An HJ Chun SH Kang K. Cascaded wx: A novel prognosis-related feature selection framework in human lung adenocarcinoma transcriptomes. Front Genet (2019) 10:662. doi: 10.3389/fgene.2019.00662 31379926 PMC6658675

[104] Choi H Na KJ. A risk stratification model for lung cancer based on gene coexpression network and deep learning. BioMed Res Int (2018) 2018:2914280. doi: 10.1155/2018/2914280 29581968 PMC5822793

[105] Amini M Hajianfar G Hadadi Avval A Nazari M Deevband MR Oveisi M. Overall survival prognostic modelling of non-small cell lung cancer patients using positron emission tomography/computed tomography harmonised radiomics features: the quest for the optimal machine learning algorithm. Clin Oncol (R Coll Radiol). (2022) 34(2):114–27. doi: 10.1016/j.clon.2021.11.014 34872823

[106] Chen W Hou X Hu Y Huang G Ye X Nie S. A deep learning- and CT image-based prognostic model for the prediction of survival in non-small cell lung cancer. Med Phys (2021) 48(12):7946–58. doi: 10.1002/mp.15302 34661294

[107] Yuan Q Cai T Hong C Du M Johnson BE Lanuti M. Performance of a machine learning algorithm using electronic health record data to identify and estimate survival in a longitudinal cohort of patients with lung cancer. JAMA Netw Open (2021) 4(7):e2114723. doi: 10.1001/jamanetworkopen.2021.14723 34232304 PMC8264641

[108] Lin T Mai J Yan M Li Z Quan X Chen X. A nomogram based on CT deep learning signature: A potential tool for the prediction of overall survival in resected non-small cell lung cancer patients. Cancer Manag Res (2021) 13:2897–906. doi: 10.2147/CMAR.S299020 PMC8019610 33833572

[109] Mukherjee P Zhou M Lee E Schicht A Balagurunathan Y Napel S. A shallow convolutional neural network predicts prognosis of lung cancer patients in multi-institutional CT-image data. Nat Mach Intell (2020) 2(5):274–82. doi: 10.1038/s42256-020-0173-6 PMC8008967 33791593

[110] Xu J Nie H He J Wang X Liao K Tu L. Using machine learning modeling to explore new immune-related prognostic markers in non-small cell lung cancer. Front Oncol (2020) 10:550002. doi: 10.3389/fonc.2020.550002 33215029 PMC7665579

[111] Wolsztynski E O'Sullivan J Hughes NM Mou T Murphy P O'Sullivan F. Combining structural and textural assessments of volumetric FDG-PET uptake in NSCLC. IEEE Trans Radiat Plasma Med Sci (2019) 3(4):421–33. doi: 10.1109/TRPMS.2019.2912433 PMC7597463 33134652

[112] Takahashi S Asada K Takasawa K Shimoyama R Sakai A Bolatkan A. Predicting deep learning based multi-omics parallel integration survival subtypes in lung cancer using reverse phase protein array data. Biomolecules (2020) 10(10). doi: 10.3390/biom10101460 PMC7603376 33086649

[113] Lai YH Chen WN Hsu TC Lin C Tsao Y Wu S. Overall survival prediction of non-small cell lung cancer by integrating microarray and clinical data with deep learning. Sci Rep (2020) 10(1):4679. doi: 10.1038/s41598-020-61588-w 32170141 PMC7069964

[114] Baek S He Y Allen BG Buatti JM Smith BJ Tong L. Deep segmentation networks predict survival of non-small cell lung cancer. Sci Rep (2019) 9(1):17286. doi: 10.1038/s41598-019-53461-2 31754135 PMC6872742

[115] Sun W Jiang M Dang J Chang P Yin FF. Effect of machine learning methods on predicting NSCLC overall survival time based on Radiomics analysis. Radiat Oncol (2018) 13(1):197. doi: 10.1186/s13014-018-1140-9 30290849 PMC6173915

[116] Chatzimichail E Matthaios D Bouros D Karakitsos P Romanidis K Kakolyris S. gamma -H2AX: A novel prognostic marker in a prognosis prediction model of patients with early operable non-small cell lung cancer. Int J Genomics (2014) 2014:160236. doi: 10.1155/2014/160236 24527431 PMC3910456

[117] Hanai T Yatabe Y Nakayama Y Takahashi T Honda H Mitsudomi T. Prognostic models in patients with non-small-cell lung cancer using artificial neural networks in comparison with logistic regression. Cancer Sci (2003) 94(5):473–7. doi: 10.1111/j.1349-7006.2003.tb01467.x PMC11160259 12824896

[118] Chen W Xu M Sun Y Ji C Chen L Liu S. Integrative predictive models of computed tomography texture parameters and hematological parameters for lymph node metastasis in lung adenocarcinomas. J Comput Assist Tomogr. (2022) 46(2):315–24. doi: 10.1097/RCT.0000000000001264 PMC8929299 35297587

[119] Lv J Chen X Liu X Du D Lv W Lu L. Imbalanced data correction based PET/CT radiomics model for predicting lymph node metastasis in clinical stage T1 lung adenocarcinoma. Front Oncol (2022) 12:788968. doi: 10.3389/fonc.2022.788968 35155231 PMC8831550

[120] Chong Y Wu Y Liu J Han C Gong L Liu X. Clinicopathological models for predicting lymph node metastasis in patients with early-stage lung adenocarcinoma: the application of machine learning algorithms. J Thorac Dis (2021) 13(7):4033–42. doi: 10.21037/jtd-21-98 PMC8339794 34422333

[121] Li Q He XQ Fan X Zhu CN Lv JW Luo TY. Development and validation of a combined model for preoperative prediction of lymph node metastasis in peripheral lung adenocarcinoma. Front Oncol (2021) 11:675877. doi: 10.3389/fonc.2021.675877 34109124 PMC8180898

[122] Zhao X Wang X Xia W Li Q Zhou L Li Q. A cross-modal 3D deep learning for accurate lymph node metastasis prediction in clinical stage T1 lung adenocarcinoma. Lung Cancer. (2020) 145:10–7. doi: 10.1016/j.lungcan.2020.04.014 32387813

[123] Liu C Huang Q Ma W Qi L Wang Y Qu T. A combination of tumor and molecular markers predicts a poor prognosis in lung adenocarcinoma. Int J Clin Exp Pathol (2019) 12(5):1690–701. PMC6947110 31933987

[124] Wang Y Deng H Xin S Zhang K Shi R Bao X. Prognostic and predictive value of three DNA methylation signatures in lung adenocarcinoma. Front Genet (2019) 10:349. doi: 10.3389/fgene.2019.00349 31105737 PMC6492637

[125] Zhao X Wang X Xia W Zhang R Jian J Zhang J. 3D multi-scale, multi-task, and multi-label deep learning for prediction of lymph node metastasis in T1 lung adenocarcinoma patients' CT images. Comput Med Imaging Graph. (2021) 93:101987. doi: 10.1016/j.compmedimag.2021.101987 34610501

[126] Ubaldi L Valenti V Borgese RF Collura G Fantacci ME Ferrera G. Strategies to develop radiomics and machine learning models for lung cancer stage and histology prediction using small data samples. Phys Med (2021) 90:13–22. doi: 10.1016/j.ejmp.2021.08.015 34521016

[127] Choi J Cho HH Kwon J Lee HY Park H. A cascaded neural network for staging in non-small cell lung cancer using pre-treatment CT. Diagnostics (Basel) (2021) 11(6). doi: 10.3390/diagnostics11061047 PMC8229025 34200270

[128] Miller HA Yin X Smith SA Hu X Zhang X Yan J. Evaluation of disease staging and chemotherapeutic response in non-small cell lung cancer from patient tumor-derived metabolomic data. Lung Cancer. (2021) 156:20–30. doi: 10.1016/j.lungcan.2021.04.012 33882406 PMC8138715

[129] Moitra D Mandal RK. Automated AJCC (7th edition) staging of non-small cell lung cancer (NSCLC) using deep convolutional neural network (CNN) and recurrent neural network (RNN). Health Inf Sci Syst (2019) 7(1):14. doi: 10.1007/s13755-019-0077-1 31406570 PMC6667593

[130] Yu L Tao G Zhu L Wang G Li Z Ye J. Prediction of pathologic stage in non-small cell lung cancer using machine learning algorithm based on CT image feature analysis. BMC Cancer. (2019) 19(1):464. doi: 10.1186/s12885-019-5646-9 31101024 PMC6525347

[131] Kirienko M Sollini M Corbetta M Voulaz E Gozzi N Interlenghi M. Radiomics and gene expression profile to characterise the disease and predict outcome in patients with lung cancer. Eur J Nucl Med Mol Imaging. (2021) 48(11):3643–55. doi: 10.1007/s00259-021-05371-7 PMC8440255 33959797

[132] Luo R Song J Xiao X Xie Z Zhao Z Zhang W. Identifying CpG methylation signature as a promising biomarker for recurrence and immunotherapy in non-small-cell lung carcinoma. Aging (2020) 12(14):14649–76. doi: 10.18632/aging.103517 PMC7425482 32723974

[133] Ahn HK Lee H Kim SG Hyun SH. Pre-treatment (18)F-FDG PET-based radiomics predict survival in resected non-small cell lung cancer. Clin Radiol (2019) 74(6):467–73. doi: 10.1016/j.crad.2019.02.008 30898382

[134] Lee B Chun SH Hong JH Woo IS Kim S Jeong JW. DeepBTS: prediction of recurrence-free survival of non-small cell lung cancer using a time-binned deep neural network. Sci Rep (2020) 10(1):1952. doi: 10.1038/s41598-020-58722-z 32029785 PMC7005286

[135] Hu J Yu H Sun L Yan Y Zhang L Jiang G. Identification of an individualized metabolism prognostic signature and related therapy regimens in early stage lung adenocarcinoma. Front Oncol (2021) 11:650853. doi: 10.3389/fonc.2021.650853 33996569 PMC8113858

[136] Kim H Goo JM Lee KH Kim YT Park CM. Preoperative CT-based deep learning model for predicting disease-free survival in patients with lung adenocarcinomas. Radiology. (2020) 296(1):216–24. doi: 10.1148/radiol.2020192764 32396042

[137] Zhao QY Liu LP Lu L Gui R Luo YW. A novel intercellular communication-associated gene signature for prognostic prediction and clinical value in patients with lung adenocarcinoma. Front Genet (2021) 12:702424. doi: 10.3389/fgene.2021.702424 34497634 PMC8419521

[138] Yoshihara K Shahmoradgoli M Martinez E Vegesna R Kim H Torres-Garcia W. Inferring tumour purity and stromal and immune cell admixture from expression data. Nat Commun (2013) 4:2612. doi: 10.1038/ncomms3612 24113773 PMC3826632

[139] Chen Y Miao S Zhao W. Identification and validation of significant gene mutations to predict clinical benefit of immune checkpoint inhibitors in lung adenocarcinoma. Am J Transl Res (2021) 13(3):1051–63. PMC8014424 33841639

[140] Peng J Zou D Gong W Kang S Han L. Deep neural network classification based on somatic mutations potentially predicts clinical benefit of immune checkpoint blockade in lung adenocarcinoma. Oncoimmunology. (2020) 9(1):1734156. doi: 10.1080/2162402X.2020.1734156 32158626 PMC7051190

[141] Li C Tian C Zeng Y Liang J Yang Q Gu F. Machine learning and bioinformatics analysis revealed classification and potential treatment strategy in stage 3-4 NSCLC patients. BMC Med Genomics (2022) 15(1):33. doi: 10.1186/s12920-022-01184-1 35193578 PMC8862473

[142] Benzekry S Grangeon M Karlsen M Alexa M Bicalho-Frazeto I Chaleat S. Machine learning for prediction of immunotherapy efficacy in non-small cell lung cancer from simple clinical and biological data. Cancers (Basel) (2021) 13(24). doi: 10.3390/cancers13246210 PMC8699503 34944830

[143] Ahn BC So JW Synn CB Kim TH Kim JH Byeon Y. Clinical decision support algorithm based on machine learning to assess the clinical response to anti-programmed death-1 therapy in patients with non-small-cell lung cancer. Eur J Cancer. (2021) 153:179–89. doi: 10.1016/j.ejca.2021.05.019 34182269

[144] Rounis K Makrakis D Papadaki C Monastirioti A Vamvakas L Kalbakis K. Prediction of outcome in patients with non-small cell lung cancer treated with second line PD-1/PDL-1 inhibitors based on clinical parameters: Results from a prospective, single institution study. PloS One (2021) 16(6):e0252537. doi: 10.1371/journal.pone.0252537 34061904 PMC8168865

[145] Jiang J Jin Z Zhang Y Peng L Zhang Y Zhu Z. Robust prediction of immune checkpoint inhibition therapy for non-small cell lung cancer. Front Immunol (2021) 12:646874. doi: 10.3389/fimmu.2021.646874 33927719 PMC8076602

[146] Trebeschi S Bodalal Z Boellaard TN Tareco Bucho TM Drago SG Kurilova I. Prognostic value of deep learning-mediated treatment monitoring in lung cancer patients receiving immunotherapy. Front Oncol (2021) 11:609054. doi: 10.3389/fonc.2021.609054 33738253 PMC7962549

[147] Yang Y Yang J Shen L Chen J Xia L Ni B. A multi-omics-based serial deep learning approach to predict clinical outcomes of single-agent anti-PD-1/PD-L1 immunotherapy in advanced stage non-small-cell lung cancer. Am J Transl Res (2021) 13(2):743–56. PMC7868825 33594323

[148] Wiesweg M Mairinger F Reis H Goetz M Kollmeier J Misch D. Machine learning reveals a PD-L1-independent prediction of response to immunotherapy of non-small cell lung cancer by gene expression context. Eur J Cancer. (2020) 140:76–85. doi: 10.1016/j.ejca.2020.09.015 33059196

[149] Arbour KC Luu AT Luo J Rizvi H Plodkowski AJ Sakhi M. Deep learning to estimate RECIST in patients with NSCLC treated with PD-1 blockade. Cancer Discovery (2021) 11(1):59–67. doi: 10.1158/2159-8290.CD-20-0419 32958579 PMC7981277

[150] Muller M Hummelink K Hurkmans DP Niemeijer AN Monkhorst K Roder J. A serum protein classifier identifying patients with advanced non-small cell lung cancer who derive clinical benefit from treatment with immune checkpoint inhibitors. Clin Cancer Res (2020) 26(19):5188–97. doi: 10.1158/1078-0432.CCR-20-0538 32631957

[151] Dercle L Fronheiser M Lu L Du S Hayes W Leung DK. Identification of non-small cell lung cancer sensitive to systemic cancer therapies using radiomics. Clin Cancer Res (2020) 26(9):2151–62. doi: 10.1158/1078-0432.CCR-19-2942 PMC9239371 32198149

[152] Khorrami M Prasanna P Gupta A Patil P Velu PD Thawani R. Changes in CT radiomic features associated with lymphocyte distribution predict overall survival and response to immunotherapy in non-small cell lung cancer. Cancer Immunol Res (2020) 8(1):108–19. doi: 10.1158/2326-6066.CIR-19-0476 PMC7718609 31719058

[153] Avanzo M Gagliardi V Stancanello J Blanck O Pirrone G El Naqa I. Combining computed tomography and biologically effective dose in radiomics and deep learning improves prediction of tumor response to robotic lung stereotactic body radiation therapy. Med Phys (2021) 48(10):6257–69. doi: 10.1002/mp.15178 PMC9753143 34415574

[154] Hosny A Parmar C Coroller TP Grossmann P Zeleznik R Kumar A. Deep learning for lung cancer prognostication: A retrospective multi-cohort radiomics study. PloS Med (2018) 15(11):e1002711. doi: 10.1371/journal.pmed.1002711 30500819 PMC6269088

[155] Li S Yang N Li B Zhou Z Hao H Folkert MR. A pilot study using kernelled support tensor machine for distant failure prediction in lung SBRT. Med Image Anal (2018) 50:106–16. doi: 10.1016/j.media.2018.09.004 PMC6237633 30266009

[156] Jayasurya K Fung G Yu S Dehing-Oberije C De Ruysscher D Hope A. Comparison of Bayesian network and support vector machine models for two-year survival prediction in lung cancer patients treated with radiotherapy. Med Phys (2010) 37(4):1401–7. doi: 10.1118/1.3352709 20443461

[157] Zhu JM Sun L Wang L Zhou TC Yuan Y Zhen X. Radiomics combined with clinical characteristics predicted the progression-free survival time in first-line targeted therapy for advanced non-small cell lung cancer with EGFR mutation. BMC Res Notes. (2022) 15(1):140. doi: 10.1186/s13104-022-06019-x 35422007 PMC9008953

[158] Tang X Li Y Yan WF Qian WL Pang T Gong YL. Machine learning-based CT radiomics analysis for prognostic prediction in metastatic non-small cell lung cancer patients with EGFR-T790M mutation receiving third-generation EGFR-TKI osimertinib treatment. Front Oncol (2021) 11:719919. doi: 10.3389/fonc.2021.719919 34660285 PMC8511497

[159] Couetoux du Tertre M Marques M McNamara S Gambaro K Hoffert C Tremblay L. Discovery of a putative blood-based protein signature associated with response to ALK tyrosine kinase inhibition. Clin Proteomics. (2020) 17:5. doi: 10.1186/s12014-020-9269-6 32055239 PMC7006423

[160] Yoo J Lee J Cheon M Woo SK Ahn MJ Pyo HR. Predictive value of (18)F-FDG PET/CT using machine learning for pathological response to neoadjuvant concurrent chemoradiotherapy in patients with stage III non-small cell lung cancer. Cancers (Basel) (2022) 14(8). doi: 10.3390/cancers14081987 PMC9031866 35454899

[161] Sepehri S Tankyevych O Iantsen A Visvikis D Hatt M Cheze Le Rest C. Accurate Tumor Delineation vs. Rough Volume of Interest Analysis for (18)F-FDG PET/CT Radiomics-Based Prognostic Modeling inNon-Small Cell Lung Cancer. Front Oncol (2021) 11:726865. doi: 10.3389/fonc.2021.726865 34733779 PMC8560021

[162] Sepehri S Tankyevych O Upadhaya T Visvikis D Hatt M Cheze Le Rest C. Comparison and fusion of machine learning algorithms for prospective validation of PET/CT radiomic features prognostic value in stage II-III non-small cell lung cancer. Diagnostics (Basel). (2021) 11(4). doi: 10.3390/diagnostics11040675 PMC8069690 33918681

[163] Kanwal B Biswas S Seminara RS Jeet C. Immunotherapy in advanced non-small cell lung cancer patients: ushering chemotherapy through the checkpoint inhibitors? Cureus (2018) 10(9):e3254. doi: 10.7759/cureus.3254 30416904 PMC6217867

[164] Xu J Zhang X Yang H Ding G Jin B Lou Y. Comparison of outcomes of tyrosine kinase inhibitor in first- or second-line therapy for advanced non-small-cell lung cancer patients with sensitive EGFR mutations. Oncotarget. (2016) 7(42):68442–8. doi: 10.18632/oncotarget.12035 PMC5356566 27637087

[165] Gao G Ren S Li A Xu J Xu Q Su C. Epidermal growth factor receptor-tyrosine kinase inhibitor therapy is effective as first-line treatment of advanced non-small-cell lung cancer with mutated EGFR: A meta-analysis from six phase III randomized controlled trials. Int J Cancer. (2012) 131(5):E822–9. doi: 10.1002/ijc.27396 22161771

[166] Ravanelli M Agazzi GM Ganeshan B Roca E Tononcelli E Bettoni V. CT texture analysis as predictive factor in metastatic lung adenocarcinoma treated with tyrosine kinase inhibitors (TKIs). Eur J Radiol (2018) 109:130–5. doi: 10.1016/j.ejrad.2018.10.016 30527295

[167] Sebastian NT Xu-Welliver M Williams TM. Stereotactic body radiation therapy (SBRT) for early stage non-small cell lung cancer (NSCLC): contemporary insights and advances. J Thorac Dis (2018) 10(Suppl 21):S2451–S64. doi: 10.21037/jtd.2018.04.52 PMC6123192 30206491

[168] Conibear J AstraZeneca UKL. Rationale for concurrent chemoradiotherapy for patients with stage III non-small-cell lung cancer. Br J Cancer. (2020) 123(Suppl 1):10–7. doi: 10.1038/s41416-020-01070-6 PMC7735212 33293671

[169] FDA Adverse Event Reporting System (FAERS) Publich Dashboard Available at: https://fis.fda.gov/sense/app/95239e26-e0be-42d9-a960-9a5f7f1c25ee/sheet/7a47a261-d58b-4203-a8aa-6d3021737452/state/analysis.

[170] Ochoa D Hercules A Carmona M Suveges D Baker J Malangone C. The next-generation Open Targets Platform: reimagined, redesigned, rebuilt. Nucleic Acids Res (2023) 51(D1):D1353–D9. doi: 10.1093/nar/gkac1046 PMC9825572 36399499

[171] Kalra A Rashdan S. The toxicity associated with combining immune check point inhibitors with tyrosine kinase inhibitors in patients with non-small cell lung cancer. Front Oncol (2023) 13:1158417. doi: 10.3389/fonc.2023.1158417 37124513 PMC10140561

[172] Katsuta Y Kadoya N Mouri S Tanaka S Kanai T Takeda K. Prediction of radiation pneumonitis with machine learning using 4D-CT based dose-function features. J Radiat Res (2022) 63(1):71–9. doi: 10.1093/jrr/rrab097 PMC8776701 34718683

[173] Kawahara D Imano N Nishioka R Ogawa K Kimura T Nakashima T. Prediction of radiation pneumonitis after definitive radiotherapy for locally advanced non-small cell lung cancer using multi-region radiomics analysis. Sci Rep (2021) 11(1):16232. doi: 10.1038/s41598-021-95643-x 34376721 PMC8355298

[174] Yu H Wu H Wang W Jolly S Jin JY Hu C. Machine learning to build and validate a model for radiation pneumonitis prediction in patients with non-small cell lung cancer. Clin Cancer Res (2019) 25(14):4343–50. doi: 10.1158/1078-0432.CCR-18-1084 30992302

[175] Cui S Luo Y Tseng HH Ten Haken RK El Naqa I. Combining handcrafted features with latent variables in machine learning for prediction of radiation-induced lung damage. Med Phys (2019) 46(5):2497–511. doi: 10.1002/mp.13497 PMC6510637 30891794

[176] Yu H Lam KO Wu H Green M Wang W Jin JY. Weighted-support vector machine learning classifier of circulating cytokine biomarkers to predict radiation-induced lung fibrosis in non-small-cell lung cancer patients. Front Oncol (2020) 10:601979. doi: 10.3389/fonc.2020.601979 33598430 PMC7883680

[177] Heilbroner SP Few R Mueller J Chalwa J Charest F Suryadevara S. Predicting cardiac adverse events in patients receiving immune checkpoint inhibitors: a machine learning approach. J Immunother Cancer (2021) 9(10). doi: 10.1136/jitc-2021-002545 PMC8491414 34607896

[178] Chen X Sheikh K Nakajima E Lin CT Lee J Hu C. Radiation versus immune checkpoint inhibitor associated pneumonitis: distinct radiologic morphologies. Oncologist. (2021) 26(10):e1822–e32. doi: 10.1002/onco.13900 PMC8488797 34251728

[179] Jain V Berman AT. Radiation pneumonitis: old problem, new tricks. Cancers (Basel). (2018) 10(7). doi: 10.3390/cancers10070222 PMC6071030 29970850

[180] Jarzebska N Karetnikova ES Markov AG Kasper M Rodionov RN Spieth PM. Scarred lung. An update on radiation-induced pulmonary fibrosis. Front Med (Lausanne). (2020) 7:585756. doi: 10.3389/fmed.2020.585756 33521012 PMC7843914

[181] Mahmood SS Fradley MG Cohen JV Nohria A Reynolds KL Heinzerling LM. Myocarditis in patients treated with immune checkpoint inhibitors. J Am Coll Cardiol (2018) 71(16):1755–64. doi: 10.1016/j.jacc.2018.02.037 PMC6196725 29567210

[182] List of Cleared or Approved Companion Diagnostic Devices (In vitro and Imaging Tools) Available at: https://www.fda.gov/medical-devices/in-vitro-diagnostics/list-cleared-or-approved-companion-diagnostic-devices-in-vitro-and-imaging-tools.

[183] Huang X Sun Y Tan M Ma W Gao P Qi L. Three-dimensional convolutional neural network-based prediction of epidermal growth factor receptor expression status in patients with non-small cell lung cancer. Front Oncol (2022) 12:772770. doi: 10.3389/fonc.2022.772770 35186727 PMC8848731

[184] Li S Luo T Ding C Huang Q Guan Z Zhang H. Detailed identification of epidermal growth factor receptor mutations in lung adenocarcinoma: Combining radiomics with machine learning. Med Phys (2020) 47(8):3458–66. doi: 10.1002/mp.14238 32416013

[185] Hong D Xu K Zhang L Wan X Guo Y. Radiomics signature as a predictive factor for EGFR mutations in advanced lung adenocarcinoma. Front Oncol (2020) 10:28. doi: 10.3389/fonc.2020.00028 32082997 PMC7005234

[186] Xiong JF Jia TY Li XY Yu W Xu ZY Cai XW. Identifying epidermal growth factor receptor mutation status in patients with lung adenocarcinoma by three-dimensional convolutional neural networks. Br J Radiol (2018) 91(1092):20180334. doi: 10.1259/bjr.20180334 30059241 PMC6319832

[187] Rios Velazquez E Parmar C Liu Y Coroller TP Cruz G Stringfield O. Somatic mutations drive distinct imaging phenotypes in lung cancer. Cancer Res (2017) 77(14):3922–30. doi: 10.1158/0008-5472.CAN-17-0122 PMC5528160 28566328

[188] Liu Y Zhou J Wu J Wang W Wang X Guo J. Development and validation of machine learning models to predict epidermal growth factor receptor mutation in non-small cell lung cancer: A multi-center retrospective radiomics study. Cancer Control. (2022) 29:10732748221092926. doi: 10.1177/10732748221092926 35417660 PMC9016531

[189] Haim O Abramov S Shofty B Fanizzi C DiMeco F Avisdris N. Predicting EGFR mutation status by a deep learning approach in patients with non-small cell lung cancer brain metastases. J Neurooncol. (2022) 157(1):63–9. doi: 10.1007/s11060-022-03946-4 35119589

[190] Wang C Xu X Shao J Zhou K Zhao K He Y. Deep learning to predict EGFR mutation and PD-L1 expression status in non-small-cell lung cancer on computed tomography images. J Oncol (2021) 2021:5499385. doi: 10.1155/2021/5499385 35003258 PMC8741343

[191] Gui D Song Q Song B Li H Wang M Min X. AIR-Net: A novel multi-task learning method with auxiliary image reconstruction for predicting EGFR mutation status on CT images of NSCLC patients. Comput Biol Med (2022) 141:105157. doi: 10.1016/j.compbiomed.2021.105157 34953355

[192] Yang X Liu M Ren Y Chen H Yu P Wang S. Using contrast-enhanced CT and non-contrast-enhanced CT to predict EGFR mutation status in NSCLC patients-a radiomics nomogram analysis. Eur Radiol (2022) 32(4):2693–703. doi: 10.1007/s00330-021-08366-y PMC8921110 34807270

[193] Song J Ding C Huang Q Luo T Xu X Chen Z. Deep learning predicts epidermal growth factor receptor mutation subtypes in lung adenocarcinoma. Med Phys (2021) 48(12):7891–9. doi: 10.1002/mp.15307 34669994

[194] Le NQK Kha QH Nguyen VH Chen YC Cheng SJ Chen CY. Machine learning-based radiomics signatures for EGFR and KRAS mutations prediction in non-small-cell lung cancer. Int J Mol Sci (2021) 22(17). doi: 10.3390/ijms22179254 PMC8431041 34502160

[195] Yin G Wang Z Song Y Li X Chen Y Zhu L. Prediction of EGFR mutation status based on (18)F-FDG PET/CT imaging using deep learning-based model in lung adenocarcinoma. Front Oncol (2021) 11:709137. doi: 10.3389/fonc.2021.709137 34367993 PMC8340023

[196] Ren M Yang H Lai Q Shi D Liu G Shuang X. MRI-based radiomics analysis for predicting the EGFR mutation based on thoracic spinal metastases in lung adenocarcinoma patients. Med Phys (2021) 48(9):5142–51. doi: 10.1002/mp.15137 34318502

[197] Dong Y Hou L Yang W Han J Wang J Qiang Y. Multi-channel multi-task deep learning for predicting EGFR and KRAS mutations of non-small cell lung cancer on CT images. Quant Imaging Med Surg (2021) 11(6):2354–75. doi: 10.21037/qims-20-600 PMC8107307 34079707

[198] Zhang B Qi S Pan X Li C Yao Y Qian W. Deep CNN model using CT radiomics feature mapping recognizes EGFR gene mutation status of lung adenocarcinoma. Front Oncol (2020) 10:598721. doi: 10.3389/fonc.2020.598721 33643902 PMC7907520

[199] Jiang X Ren M Shuang X Yang H Shi D Lai Q. Multiparametric MRI-based radiomics approaches for preoperative prediction of EGFR mutation status in spinal bone metastases in patients with lung adenocarcinoma. J Magn Reson Imaging. (2021) 54(2):497–507. doi: 10.1002/jmri.27579 33638577

[200] Rossi G Barabino E Fedeli A Ficarra G Coco S Russo A. Radiomic detection of EGFR mutations in NSCLC. Cancer Res (2021) 81(3):724–31. doi: 10.1158/0008-5472.CAN-20-0999 33148663

[201] Mu W Jiang L Zhang J Shi Y Gray JE Tunali I. Non-invasive decision support for NSCLC treatment using PET/CT radiomics. Nat Commun (2020) 11(1):5228. doi: 10.1038/s41467-020-19116-x 33067442 PMC7567795

[202] Park YW An C Lee J Han K Choi D Ahn SS. Diffusion tensor and postcontrast T1-weighted imaging radiomics to differentiate the epidermal growth factor receptor mutation status of brain metastases from non-small cell lung cancer. Neuroradiology. (2021) 63(3):343–52. doi: 10.1007/s00234-020-02529-2 32827069

[203] Liu Q Sun D Li N Kim J Feng D Huang G. Predicting EGFR mutation subtypes in lung adenocarcinoma using (18)F-FDG PET/CT radiomic features. Transl Lung Cancer Res (2020) 9(3):549–62. doi: 10.21037/tlcr.2020.04.17 PMC7354146 32676319

[204] Nair JKR Saeed UA McDougall CC Sabri A Kovacina B Raidu BVS. Radiogenomic models using machine learning techniques to predict EGFR mutations in non-small cell lung cancer. Can Assoc Radiol J (2021) 72(1):109–19. doi: 10.1177/0846537119899526 32063026

[205] Li X Yin G Zhang Y Dai D Liu J Chen P. Predictive power of a radiomic signature based on (18)F-FDG PET/CT images for EGFR mutational status in NSCLC. Front Oncol (2019) 9:1062. doi: 10.3389/fonc.2019.01062 31681597 PMC6803612

[206] Zhao W Yang J Ni B Bi D Sun Y Xu M. Toward automatic prediction of EGFR mutation status in pulmonary adenocarcinoma with 3D deep learning. Cancer Med (2019) 8(7):3532–43. doi: 10.1002/cam4.2233 PMC6601587 31074592

[207] Li XY Xiong JF Jia TY Shen TL Hou RP Zhao J. Detection of epithelial growth factor receptor (EGFR) mutations on CT images of patients with lung adenocarcinoma using radiomics and/or multi-level residual convolutionary neural networks. J Thorac Dis (2018) 10(12):6624–35. doi: 10.21037/jtd.2018.11.03 PMC6344758 30746208

[208] Wang S Shi J Ye Z Dong D Yu D Zhou M. Predicting EGFR mutation status in lung adenocarcinoma on computed tomography image using deep learning. Eur Respir J (2019) 53(3). doi: 10.1183/13993003.00986-2018 PMC6437603 30635290

[209] Li Y Lu L Xiao M Dercle L Huang Y Zhang Z. CT slice thickness and convolution kernel affect performance of a radiomic model for predicting EGFR status in non-small cell lung cancer: A preliminary study. Sci Rep (2018) 8(1):17913. doi: 10.1038/s41598-018-36421-0 30559455 PMC6297245

[210] Sha L Osinski BL Ho IY Tan TL Willis C Weiss H. Multi-field-of-view deep learning model predicts nonsmall cell lung cancer programmed death-ligand 1 status from whole-slide hematoxylin and eosin images. J Pathol Inform. (2019) 10:24. doi: 10.4103/jpi.jpi_24_19 31523482 PMC6669997

[211] Wang C Ma J Shao J Zhang S Li J Yan J. Non-invasive measurement using deep learning algorithm based on multi-source features fusion to predict PD-L1 expression and survival in NSCLC. Front Immunol (2022) 13:828560. doi: 10.3389/fimmu.2022.828560 35464416 PMC9022118

[212] Jiang Z Dong Y Yang L Lv Y Dong S Yuan S. CT-based hand-crafted radiomic signatures can predict PD-L1 expression levels in non-small cell lung cancer: a two-center study. J Digit Imaging. (2021) 34(5):1073–85. doi: 10.1007/s10278-021-00484-9 PMC8554954 34327623

[213] Mu W Jiang L Shi Y Tunali I Gray JE Katsoulakis E. Non-invasive measurement of PD-L1 status and prediction of immunotherapy response using deep learning of PET/CT images. J Immunother Cancer (2021) 9(6). doi: 10.1136/jitc-2020-002118 PMC8211060 34135101

[214] Tian P He B Mu W Liu K Liu L Zeng H. Assessing PD-L1 expression in non-small cell lung cancer and predicting responses to immune checkpoint inhibitors using deep learning on computed tomography images. Theranostics. (2021) 11(5):2098–107. doi: 10.7150/thno.48027 PMC7797686 33500713

[215] Zhu Y Liu YL Feng Y Yang XY Zhang J Chang DD. A CT-derived deep neural network predicts for programmed death ligand-1 expression status in advanced lung adenocarcinomas. Ann Transl Med (2020) 8(15):930. doi: 10.21037/atm-19-4690 32953730 PMC7475404

[216] Chang C Sun X Wang G Yu H Zhao W Ge Y. A machine learning model based on PET/CT radiomics and clinical characteristics predicts ALK rearrangement status in lung adenocarcinoma. Front Oncol (2021) 11:603882. doi: 10.3389/fonc.2021.603882 33738250 PMC7962599

[217] Song Z Liu T Shi L Yu Z Shen Q Xu M. The deep learning model combining CT image and clinicopathological information for predicting ALK fusion status and response to ALK-TKI therapy in non-small cell lung cancer patients. Eur J Nucl Med Mol Imaging. (2021) 48(2):361–71. doi: 10.1007/s00259-020-04986-6 32794105

[218] Ma DN Gao XY Dan YB Zhang AN Wang WJ Yang G. Evaluating solid lung adenocarcinoma anaplastic lymphoma kinase gene rearrangement using noninvasive radiomics biomarkers. Onco Targets Ther (2020) 13:6927–35. doi: 10.2147/OTT.S257798 PMC7371989 32764984

[219] Song L Zhu Z Mao L Li X Han W Du H. Clinical, Conventional CT and radiomic feature-based machine learning models for predicting ALK rearrangement status in lung adenocarcinoma patients. Front Oncol (2020) 10:369. doi: 10.3389/fonc.2020.00369 32266148 PMC7099003

[220] Zhang T Xu Z Liu G Jiang B de Bock GH Groen HJM. Simultaneous identification of EGFR,KRAS,ERBB2, and TP53 mutations in patients with non-small cell lung cancer by machine learning-derived three-dimensional radiomics. Cancers (Basel) (2021) 13(8). doi: 10.3390/cancers13081814 PMC8070114 33920322

[221] Sadhwani A Chang HW Behrooz A Brown T Auvigne-Flament I Patel H. Comparative analysis of machine learning approaches to classify tumor mutation burden in lung adenocarcinoma using histopathology images. Sci Rep (2021) 11(1):16605. doi: 10.1038/s41598-021-95747-4 34400666 PMC8368039

[222] Wang J Chen P Su M Zhong G Zhang S Gou D. Integrative modeling of multiomics data for predicting tumor mutation burden in patients with lung cancer. BioMed Res Int (2022) 2022:2698190. doi: 10.1155/2022/2698190 35097114 PMC8794677

[223] He B Dong D She Y Zhou C Fang M Zhu Y. Predicting response to immunotherapy in advanced non-small-cell lung cancer using tumor mutational burden radiomic biomarker. J Immunother Cancer (2020) 8(2). doi: 10.1136/jitc-2020-000550 PMC7342823 32636239

[224] Wang M Wang S Trapani JA Neeson PJ. Challenges of PD-L1 testing in non-small cell lung cancer and beyond. J Thorac Dis (2020) 12(8):4541–8. doi: 10.21037/jtd-2019-itm-010 PMC7475552 32944371

[225] Taha T Khoury R Brenner R Nasrallah H Shofaniyeh I Yousef S. Treatment of rare mutations in patients with lung cancer. Biomedicines (2021) 9(5). doi: 10.3390/biomedicines9050534 PMC8151457 34064757

[226] Simarro J Murria R Perez-Simo G Llop M Mancheno N Ramos D. Development, implementation and assessment of molecular diagnostics by next generation sequencing in personalized treatment of cancer: experience of a public reference healthcare hospital. Cancers (Basel) (2019) 11(8). doi: 10.3390/cancers11081196 PMC6721584 31426418

[227] Breiman L. Statistical modeling: the two cultures. Stat Science. (2001) 16(3):199–215.

[228] Climente-Gonzalez H Azencott CA Kaski S Yamada M. Block HSIC Lasso: model-free biomarker detection for ultra-high dimensional data. Bioinformatics (2019) 35(14):i427–i35. doi: 10.1093/bioinformatics/btz333 PMC6612810 31510671

[229] Plana D Shung DL Grimshaw AA Saraf A Sung JJY Kann BH. Randomized clinical trials of machine learning interventions in health care: A systematic review. JAMA Netw Open (2022) 5(9):e2233946. doi: 10.1001/jamanetworkopen.2022.33946 36173632 PMC9523495

[230] Weissler EH Naumann T Andersson T Ranganath R Elemento O Luo Y. The role of machine learning in clinical research: transforming the future of evidence generation. Trials. (2021) 22(1):537. doi: 10.1186/s13063-021-05489-x 34399832 PMC8365941

[231] Artificial Intelligence and Machine Learning in Software as a Medical Device Available at: https://www.fda.gov/medical-devices/software-medical-device-samd/artificial-intelligence-and-machine-learning-software-medical-device.

[232] Liu X Cruz Rivera S Moher D Calvert MJ Denniston AK Spirit AI. Reporting guidelines for clinical trial reports for interventions involving artificial intelligence: the CONSORT-AI extension. Nat Med (2020) 26(9):1364–74. doi: 10.1038/s41591-020-1034-x PMC7598943 32908283

